# Transplanted gene-modified placental cells boost FVIII activity in pediatric sheep without eliciting immunity, toxicity, or adverse events

**DOI:** 10.3389/fimmu.2025.1716950

**Published:** 2026-01-07

**Authors:** Brady Trevisan, Martin Rodriguez, Ritu Ramamurthy, Sunil George, Oluwaseun O. Babatunde, Jacqueline Dizon, Jordan Shields, Shannon Lankford, Denise Schwahn, Michael Gautreaux, Andrew Farland, John Owen, Anthony Atala, Christopher B. Doering, H. Trent Spencer, Christopher D. Porada, Graça Almeida-Porada

**Affiliations:** 1 Fetal Research and Therapy Program, Wake Forest Institute for Regenerative Medicine, Wake Forest University School of Medicine, Winston Salem, NC, United States; 2 Aflac Cancer and Blood Disorders Center, Children’s Healthcare of Atlanta and Department of Pediatrics, Emory University, Atlanta, GA, United States; 3 Zoetis Veterinary Medical Research and Development, Kalamazoo, MI, United States; 4 Human Leukocyte Antigen (HLA)/Immunogenetics and Immunodiagnostics Laboratories, Wake Forest University School of Medicine, Winston Salem, NC, United States; 5 Special Hematology Laboratory, Wake Forest University School of Medicine, Winston Salem, NC, United States

**Keywords:** hemophilia A, FVIII, gene therapy, mesenchymal stromal cell, transplantation, placental cells, exosomes, FVIII inhibitors

## Abstract

**Background:**

The current standard of care for Hemophilia A (HA), a hereditary bleeding disorder caused by mutations in the Factor VIII (F8) gene, include FVIII replacement proteins, engineered clotting factors, and a broad array of new therapeutics including antibodies and gene therapy. These therapies allow persons with HA (PHA) to have near normal life expectancies, but the burden of disease continues to be high, with 30% of PHA developing FVIII inhibitors, considerably increasing the risk of morbidity and mortality.

**Objective:**

The present study tested the ability of human placental cells (PLC), transduced with a lentivector encoding a codon-optimized, bioengineered FVIII transgene (mcoET3) (PLC-mcoET3) to increase FVIII activity levels after administration to pediatric large animals. In addition, we determined whether administration of PLC-mcoET3 would induce inhibitor formation, and defined how the immune response to infused human FVIII (hFVIII) or ET3 proteins differed from that of administration of PLC-mcoET3.

**Methods:**

Pediatric sheep at 8–12 months of age were used in this study. PLC-mcoET3 providing 20 IU/kg of ET3/infusion/sheep were administered intravenously (IV) or intraperitoneally (IP), and control groups received the same dose/kg of purified recombinant ET3 or human full-length recombinant FVIII protein (hFVIII). Plasma FVIII activity, presence of anti-FVIII/ET3 humoral or cellular immune responses, and immunologic responses using a multiplexed gene expression panel were assessed.

**Results and conclusion:**

Data show that while intravenous (IV) infusion of ET3 or hFVIII to pediatric sheep results in a high level of inhibitory antibodies, administration of PLC-mcoET3 IV is safe, and resulted in increased plasma FVIII activity for at least 15 weeks without the formation of anti-ET3/FVIII inhibitory antibodies.

## Introduction

Hemophilia A (HA) is an X-linked genetic disorder caused by mutations in the Factor VIII (F8) gene, resulting in the lack of functional clotting protein Factor VIII (FVIII) (1).

The current standard of care for severe HA consists of the prophylactic infusion of plasma-derived or recombinant FVIII products. Although replacement FVIII therapy allows persons with HA (PHA) to have near normal life expectancies, the burden of disease continues to be high (2), and 30% of PHA develop anti-FVIII neutralizing antibodies (FVIII inhibitors), making replacement therapy ineffective and considerably increasing the risk of morbidity and mortality (3). Care in HA was revolutionized with the approval of emicizumab, a bispecific antibody that binds to both FIXa and FX, bringing them into close enough proximity to enable FX to be activated by FIXa, thereby bypassing the need for FVIII as a cofactor. Emicizumab prophylaxis has significantly slowed accumulation of factor exposure days and has shown continued effective prophylaxis in the case of inhibitor development. However, while emicizumab has proven to be markedly effective at reducing the frequency of bleeding events in PHA, management of trauma and surgeries still requires additional treatment (often FVIII), and breakthrough bleeds still occur with some frequency, especially within the joints (4–6).

PHA who develop inhibitors are extremely difficult to manage, and one of the therapeutic options is administration of very high doses of FVIII to induce immune tolerance (7–9). However, this approach is not effective in at least 1/3 of PHA, and the treatment cost, which is already expensive for PHA without inhibitors, triples for those with inhibitors (7–9). RNA interference therapeutics, FVIIa variants, and FVIII-bypassing agents can circumvent the need for FVIII products and can be used to overcome inhibitors and restore hemostasis. Nevertheless, none of these products are ideal, and some have caused severe side effects, including thrombotic complications (10).

Thus, new therapeutic approaches which can provide long-lasting therapeutic effect, without inducing inhibitors in those who are susceptible, are urgently needed for the management of PHA.

We have recently reported that human placental cells (PLC) are an excellent cell platform to produce and secrete FVIII *in vivo* when transduced with a lentivector encoding a myeloid codon-optimized (mco), bioengineered human/porcine hybrid FVIII transgene (ET3) (PLC-mcoET3) (11–13). In addition, we have shown that *in utero* transplantation (IUTx) of PLC-mcoET3 into fetal sheep, at the human equivalent of 16–18 gestation weeks, resulted in considerable elevation in plasma FVIII levels that persisted for >3 years post-IUTx (14). Since PLC have inherent immunomodulatory properties (15) and they constitutively produce vWF (16), we reasoned that postnatal administration of PLC-mcoET3 to deliver FVIII/ET3 protein would render FVIII delivery more effective and less immunogenic. In plasma, FVIII circulates tightly bound to von Willebrand factor (vWF), and the interaction between vWF and FVIII plays a critical role in the function, stability, and clearance of FVIII protein (17). Changes in vWF concentration affect FVIII levels (18), as the half-life of FVIII in the absence of vWF is approximately 3 hours, while in the presence of vWF it is 12 hours (19). The association of vWF with FVIII also plays an immuno-protective role by masking FVIII immunogenic epitopes, preventing the uptake of FVIII by antigen-presenting cells (APC) and by modulating FVIII peptide presentation in APC (20, 21). The present study tested the ability of PLC-mcoET3 to increase plasma FVIII activity levels when administrated to pediatric wild-type sheep, determined whether this method of administration could avoid the risk of inhibitor formation to this immunologically “foreign” FVIII molecule, and defined how the immune response to infusion of ET3 or human FVIII (hFVIII) proteins differed from that following administration of ET3-expressing PLC. Data show that while intravenous (IV) infusion of ET3 or hFVIII to pediatric sheep results in high levels of inhibitory antibodies, IV administration of PLC-mcoET3 results in increased plasma FVIII activity for at least 15 weeks without the formation of anti-ET3/FVIII inhibitory antibodies.

## Materials and methods

### Study design

The objective of this study was to investigate whether administration of ET3 protein through ET3-secreting cells, instead of the purified protein formulations of ET3 or FVIII, would prevent formation of FVIII inhibitory antibodies. All animal procedures were performed in accordance with Wake Forest University Health Sciences IACUC guidelines. Euthanasia was performed using Euthasol (86–100 mg/kg; I.V.) after sedation with Dexmedetomidine (0.005–0.03 mg/kg; I.V.). Two to five minutes after Euthasol administration, the animal is examined to ensure absence of ocular reflex, heartbeat, and respiration. If present, additional Euthasol will be administered until the absence of ocular reflex, heartbeat, and respiratory movements is confirmed. To determine the animal group size required to achieve sufficient power to detect statistically significant differences between the animals boosted with protein *vs.* those dosed with PLC-mcoET3 with respect to inhibitor induction, we used a binary outcome superiority trial (22), set to achieve a 95% chance of detecting, as significant at the 2.5% level, a decrease in the incidence of inhibitor formation from 90% in the protein-dosed group to 5% in the PLC-mcoET3-dosed group. The following formula was used to perform these calculations: n = f(α/2, β) × [p_1_ × (100 − p_1_) + p_2_ × (100 − p_2_)]/(p_2_ − p_1_)^2^, where p_1_ and p_2_ are the percent ‘success’ in the control and experimental group respectively, and f(α, β) = [Φ^-1^(α) + Φ^-1^(β)]^2^, where Φ^-1^ is the cumulative distribution function of a standardized normal deviate. Adjustment for cross-overs based on formula: n_adj_ = n × 10,000/(100 - c_1_ - c_2_)^2^, where c_1_ and c_2_ are the percent cross-over in the protein-dosed and PLC-mcoET3-dosed group, respectively. These calculations confirmed that 3 animals per group were sufficient to achieve the desired statistical power. A total of 12 pediatric sheep at 8–12 months of age (corresponds to 12–18 years in humans) were used in this study. Group 1 (n=3) received PLC-mcoET3 producing 6±0.4 IU/10^6^ cells/24hr of ET3 at a dose of 4x10^6^±1.3x10^6^ cells/kg, once per week for 3 weeks, IV. The doses of PLC-mcoET3 infused were calculated to provide each animal with a total dose of 60 IU/kg of ET3 (20 IU/kg of ET3/infusion). Group 2 (n=3) received a single total dose of ≈60 IU/kg/10^6^Cells/24hr intraperitoneally (IP), the preferred route of administration in prenatal studies (23). Group 3 (n=3) and Group 4 (n=3) received, for 5 weeks, weekly IV injections of 20IU/kg of purified recombinant ET3 (12) or clinical-grade human full-length recombinant FVIII protein (hFVIII), respectively (**Supplementary Figure 1**). The clinical human FVIII product we elected to use was Kogenate^®^ FS, which is a full-length recombinant human FVIII. While the presence of the B domain introduces an element absent in the ET3 protein, Kogenate was the only recombinant human FVIII product (at the time these studies were initiated) that is produced from a baby hamster kidney (BHK) cell line (24) – the same cell type in which the recombinant ET3 protein is produced. As clinical FVIII inhibitors are most frequently directed against domains of the FVIII protein other than the B domain (25–29), we did not feel this difference would result in a significant difference in the potential immunogenicity of the two FVIII proteins we infused. Moreover, as a number of studies have provided evidence that glycosylation of FVIII likely plays a key role in the differing incidence of inhibitors following administration of plasma-derived versus recombinant human FVIII products, which are produced in a variety of different cell lines (which are usually non-human) (30–34), we felt that it was more important to use a human recombinant protein produced in the same cell line as the ET3 protein than to employ a B domain deleted product.

Blood was collected from all animals prior to product(s) administration at day 0, weekly for 5 consecutive weeks, and at weeks 7, 10, and 15. Plasma and peripheral blood mononuclear cells (PBMC) were isolated from the whole blood at each of these timepoints and used to determine plasma FVIII activity, presence of anti-FVIII/ET3 humoral or cellular immune responses within these animals, and for evaluation of immunologic responses using a multiplexed gene expression panel (NanoString Technologies). Tissues collected at the end of study from these animals were evaluated for PLC-mcoET3 engraftment and gene expression by RNA-seq. In addition, 3 of the animals that received ET3 or FVIII protein and developed inhibitory antibodies were treated at 14–23 months after the administration of ET3 or FVIII protein with human PLC-mcoET3, producing 3.6 IU/10^6^cells/24hr of FVIII/ET3, in 3 consecutive weeks (20IU/kg/24h) to reach a total dose of 60IU/kg/24h dose, to determine whether IV administration of PLC-mcoET3 could overcome the presence of pre-existing inhibitors. In all studies evaluating product efficacy, safety, and FVIII/ET3 immunological responses, tests were run by individuals who were either blinded to animal treatment group and/or by unbiased evaluators.

### Isolation, culture, and transduction of placental cells

Human placental cells (PLC) were isolated, cultured and transduced with a lentiviral vector encoding mcoET3, a human B domain-deleted, myeloid-codon-optimized, bioengineered FVIII transgene containing high expression elements from porcine FVIII (35). They were then analyzed for FVIII production, phenotype, viability/function, genomic stability, and immunogenicity, as previously described (11). scRNAseq analysis demonstrated >90% transduction efficiency in PLC used for this study.

Prior to administration, PLC-mcoET3 were expanded in CellSTACK chambers (Corning) to the required numbers for transplantation, harvested, counted, and checked for viability using a Countess 3 machine and slide (ThermoFisher Scientific). After washing and centrifugation, PLC-mcoET3 were resuspended in Plasma-Lyte A (Baxter International) and thoroughly mixed by pipetting. PLC-mcoET3 were then loaded into a syringe through a 16-gauge needle, and an extra 1mL of air was aspirated into the syringe. The needle was removed from the syringe, and the cell-containing syringe was sealed in a sterile package. During transportation to the site of transplant, the syringe was frequently rotated end-over-end to prevent the cells from clumping.

### Production and purification of ET3 protein

Stable expression of ET3 from baby hamster kidney-derived (BHK-M) cells was performed as described previously (36). In brief, BHK-M cells were transfected with a plasmid construct encoding ET3, using Lipofectin™ according to the manufacturer’s instructions (ThermoFisher Scientific, Waltham, MA). Transfected cells were cultured for 10 days in DMEM/F-12 containing 10% fetal bovine serum, 100 units/ml penicillin, 100 μg/ml streptomycin, and 500 μg/ml Geneticin. Geneticin-resistant clones were seeded onto 2-cm^2^ wells, and grown to 80–90% confluence before being switched to 1 ml of AIM V serum-free medium (ThermoFisher Scientific). Cells were cultured in serum-free medium for 24 h before measurement of secreted fVIII coagulant activity as described below. ET3 was purified from BHK-M supernatants using a two-step ion-exchange chromatography procedure as described previously for the purification of recombinant human fVIII and recombinant porcine fVIII (36), with the following modifications. Conditioned medium containing ET3 was loaded onto a 2.5 x 19.5-cm SP-Sepharose Fast Flow column equilibrated at 25 °C in 0.18 M NaCl, 20 mM HEPES, 5 mM CaCl_2_, 0.01% Tween 80, pH 7.4. ET3 was eluted using a linear 0.18–0.7 M NaCl gradient in the same buffer. ET3-containing fractions were identified by SDS-PAGE and one-stage and two-stage activation quotient coagulation assays in a ST art Coagulation Instrument (Diagnostica Stago, Asnieres, France) using human fVIII-deficient plasma as substrate as described previously (37). The activation quotient is defined as the activity in the two-stage assay divided by the activity in the one-stage assay.

### Administration of therapeutic products

To deliver the PLC-mcoET3 through the intraperitoneal (IP) route, the recipient sheep were fasted for 24 hours, and water was removed the night before the transplant. The sheep were anesthetized with ketamine (2mg/kg). Upon achieving an appropriate plane of sedation/anesthesia, the sheep was laid on its side, and an electric clipper was used to remove the wool from the abdominal area. The area was then cleaned with a chlorohexidine scrub (Aspen Veterinary Supplies), and ultrasound gel was applied to the area. An echogenic, laser-etched Quincke-tip needle (Havel’s, 6PTC22) was then inserted percutaneously into the peritoneal cavity, under continuous ultrasound visualization. Upon successful placement of the needle tip within the peritoneal cavity, the stylet was removed, and the syringe containing the PLC-mcoET3 was gently screwed onto the echogenic needle. The entire cell suspension was then slowly expelled into the peritoneal cavity under continuous ultrasound visualization, after which the needle was removed. The animal was then monitored until it had fully regained consciousness.

To deliver the PLC-mcoET3 through the intravenous (IV) route, a 22-gauge needle attached to the syringe containing the cells was used to administer PLC-mcoET3 into the left jugular vein of the sheep. Pressure was then applied for 30 seconds to ensure that any bleeding had stopped. Bolus infusions of hFVIII and ET3 proteins were administered in the same fashion as the IV transplant of the PLC-mcoET3. All animals were closely monitored for 5 days after each injection.

### Coagulation assays for factor VIII activity

Plasma FVIII activity was determined by activated partial thromboplastin time (aPTT) one-stage assay that was performed by the Atrium Health Wake Forest Baptist Medical Center Special Hematology Laboratory in accordance with standard clinical procedures, using a Top 300 CTS clinical coagulometer (Instrumentation Laboratories). All tests were run with investigators blinded to animal treatment group. For each new set of reagents, values were normalized to control samples from previous runs to account for variation between runs.

### Anti-mcoET3 IgM and IgG antibody enzyme-linked immunosorbent assay (s)

High-binding plates (Corning, 9018) were coated with ET3 protein as previously described (38). Sheep plasma samples were serially diluted starting at 1:20 dilution, and antibody binding was detected with anti-ovine IgG: AP (Bio-Rad, STAR88A) or anti-ovine IgM: AP (Abcam, AB112761), and p-nitrophenyl-phosphate (Bio-Rad, 1721063). The antibody titer was defined as the dilution of plasma with an absorbance value >2 SD above the mean OD from control sheep plasma (n=4).

### Antigen-specific T_h1_ and T_h2_ Cell ELISpot

Reactive memory T_h1_ and T_h2_ responses to ET3 were determined by IFN-γ (MabTech, 3119-4APW-2) and IL-4 (MabTech, 3118-2A) ELISpot assays, as previously described (39, 40). A positive response was defined by a Stimulation Index >2, and > 10 SFU/2x10^5^ PBMC.

### One-way mixed lymphocyte reactions

T cell proliferation was evaluated by 5-bromo-2’-deoxyuridine (BrdU) ELISA (Roche,12352200). *Responder cells* consisted of PBMC from treated and untreated (control) animals, and *stimulator cells* were mitomycin C-treated same-donor non-transduced PLC, same-donor PLC-mcoET3, or third-party human PBMC. The stimulation index (SI) was calculated based on typical SI criteria for MLR cultures, where: SI=(Stimulator+ Responder)/(Responder).

### Luminex ID assay for detection of anti-HLA class I antibodies

Detection of IgG antibodies specific to HLA Class I and Class II were detected by the HLA/Immunogenetics and Immunodiagnostic Laboratory using Luminex assays according to the manufacturer’s specifications (Immucor, 628200).

### Liver enzyme tests and white blood cell counts

Aspartate aminotransferase (AST) and alanine aminotransferase (ALT) assays were performed blinded by the Cornell University Animal Health Diagnostic Center. Normal values for AST and ALT in sheep are < 280 and < 50U/L, respectively. Whole blood was used to determine WBC/mL using the LeukoCheck test kit (Biomedical Polymers, 21243285).

### RT-qPCR and dPCR for analysis of cell engraftment

To quantify levels of PLC-mcoET3 present in different tissues, RT-qPCR was performed with the Applied Biosystems QuantStudio 6 Flex Real-Time PCR system, using primers specific for mcoET3 and sheep GAPDH as previously reported in detail (14). Percentage of engraftment was determined by comparing ΔCt values to a standard curve consisting of increasing percentages of PLC-mcoET3 (0, 0.01, 0.1, 1, 5, and 10%) in sheep stromal cells. RNA isolated from the same tissues of non-transplanted animals served as controls and yielded no amplification. dPCR was performed via the absolute Q™ digital PCR. Primers and probes targeting human Alu-specific sequences (41) and sheep-specific RPL13A sequences (Genscript) were custom -designed based on TaqMan chemistry using the following primers: Forward primer (101 F), 5′-GGTGAAACCCCGTCTCTACT-3′ Reverse primer (206 R), 5′-GGTTCAAGCGATTCTCCTGC-3′. The sequence of the hydrolysis probe (144RH) was 5′-CGCCCGGCTAATTTTTGTAT-3′. The probe 144RH was labeled with the fluorescent reporter 6-carboxy-fluorescein (6-FAM) at the 5′ end and the fluorescent quencher Black Hole Quencher 1 (BHQ1) at the 3′ end. All oligos were custom-synthesized by Integrated DNA Technologies, Inc. (Coralville, Iowa, USA). Each reaction contained 200nM of Alu and RPL13A primers and 250nM of their respective probes in a final concentration of 1X Absolute Q™ DNA Digital PCR Master Mix (ThermoFisher Scientific, A52490). A final reaction volume of 9ul was achieved by adding nuclease-free PCR-grade water (ThermoFisher Scientific, AM9935). The reaction mixture was loaded into the Applied Biosystems™ QuantStudio™ MAP16 Digital PCR Plate (ThermoFisher Scientific, A52865), followed by an overlay of 15μl of isolation buffer (ThermoFisher Scientific, A52730). The prepared plate was then loaded on the QuantStudio Absolute Q dPCR System (Applied Biosystems, Foster City, CA) and run in three individual separate experiments with the following cycling parameters: a pre-heat activation cycle of 96°C for 10 minutes, followed by 40 cycles of 96°C for 5 s and 60°C for 15 s.

### Tissue histopathology and immunohistochemistry

To determine the location of PLC-mcoET3 in tissues, IHC staining was performed on paraffin-embedded tissues collected from animals euthanized at 7–18 months post-W0. Liver and lung sections were stained using a human-specific antibody to the nuclear antigen, Ku80 (Cell Signaling Technologies, 2753S) and detected with a goat anti-rabbit IgG Alexa-Fluor 594 (Life Technologies, A11012). Confocal images were acquired with an Olympus Fluoview FV1000 confocal microscope and an Olympus UPlanFLN- 40x/1.30 oil objective. An outside specialized veterinary pathologist performed gross anatomical examination to determine the presence of therapy-related morphologic abnormalities, and evaluated hematoxylin and eosin (H&E)-stained slides of liver, lungs, spleen, thymus, intestine, and brain to identify potential histopathologic alterations.

### NanoString analysis

RNA was extracted from PBMC from animals in each treatment group at weeks 0 and 5 using an RNeasy Plus Mini Kit (Qiagen, 74034). RNA quality was confirmed with the RNA Nano 6000 kit (Agilent Technologies, 5067-1511). RNA was hybridized to a custom NanoString CodeSet by first adding 70μL of hybridization buffer to the Reporter CodeSet, 8μL of MasterMix, and 5μL of sample to each tube in a 12-tube strip, followed by adding 2μL of Capture Probeset. These tube strips were then incubated for 16 hours at 70˚C. After hybridization, 30μL of each of these samples were then added to individual wells of a NanoString cartridge, and the cartridge was sealed. The cartridge was run on a NanoString Sprint Profiler, and the data were analyzed using nSolver 4.0 (NanoString Technologies).

### NanoString multiplex gene expression analysis

A multiplex gene expression analysis, with 165 gene targets involved in antigen-specific immune cell signaling, was performed to identify differences in response profiles between the various treatment groups (see **Supplementary Table 1** for gene target list). Total mRNA from PBMC was evaluated for changes in expression of the target genes at Weeks 0 and 5 post-treatment using the NanoString Technologies nCounter Sprint Profiler, in triplicates for all animals. After acquiring raw counts, data were analyzed using NanoString nSolver 4.0 software. Differential expression was calculated by considering each Group’s Week 0 as baseline. Regarding normalization parameters, CodeSet content was normalized using HPRT1 as the reference gene, and for CodeSet calibration, a technical replicate was used across RLF sets. The advanced analysis settings were used to perform multi-RLF experiment, selecting Differential Expression, Pathway Scoring, and Cell Type analyses. Statistical significance of the observed fold-change in expression was calculated with a Benjamini-Hochberg false discovery rate correction.

### RNA-seq analysis using next generation sequencing

Complete transcriptome RNA-seq libraries were created using the QIAseq UPXome RNA Lib Kit HMR (Qiagen, 334705) following the manufacturer’s standard protocol. In brief, total RNA was subjected to ribosomal RNA (rRNA) suppression using QIAseq FastSelect rRNA removal kits HMR, and was then reverse transcribed into cDNA using random hexamers and oligo-dT primers for 90 minutes at 42°C. During cDNA synthesis, each sample received a unique sample ID that allowed for cDNA pooling. All the subsequent library construction steps of pooled samples was done in a single tube to minimize batch affects. Following cDNA pooling, 2 rounds of bead selection/reaction clean-up was performed with QIAseq beads. The cDNA was then amplified with unique dual-sample indexes using the QIAseq UX 12 Index Kit IL UDI (Qiagen,331801). Completed libraries were then purified using 2 rounds of bead-based cleanup with QIAseq beads. Final libraries were quality controlled using an Agilent Bioanalyzer, quantitated by real-time qPCR using the QIAseq Library Quant Assay Kit (Qiagen, 333314), and sequenced using an Illumina Next-Seq 500.

### Data analysis: demultiplexing, read mapping

FASTQ files were uploaded to the QIAGEN RNA-seq Analysis Portal: (https://geneglobe.qiagen.com/us/analyze/), and aligned to *Homo sapiens* (GRCh38.noalt.103) with analysis workflow version 1.0.:

(https://resources.qiagenbioinformatics.com/manuals/rnaanalysisportal/current/index.php?manual=QIAseq_UPXome_RNA_Lib_Kit.html)

RNA-seq libraries were aligned to *Homo sapiens* libraries after eliminating any possible cross-reactivity with ovine transcripts due to sequence homology between the human and sheep transcripts. We then performed pairwise comparisons between the transcripts present in transduced PLC-mcoET3 prior to transplant, with human transcripts known to be expressed in specific cell types within each of the different organs.

### Exosome isolation and sequencing

PLC-mcoET3 were grown to 70-80% confluency using exosome-depleted FBS (ExoFBS). Bulk cell media was collected and centrifuged at 300xg at 4°C for 10 min to pellet large dead cells, supernatant was collected, and the media centrifuged again at 2000xg at 4°C for 20 min. Supernatants were transferred to clean tubes, stored at -80°C, and shipped on dry ice to System Biosciences (SBI) for exosome isolation and sequencing. Exosome isolation was performed using SBI’s ExoQuick precipitation method. Exosomal RNA was extracted and quantified by Agilent Bioanalyzer Small RNA Assay. Next-Gen Sequencing (NGS) libraries were be prepared and sequenced on an Illumina Hi-Seq instrument with 150bp paired-end reads at an approximate depth of 10–15 million reads per sample. Bioinformatic analysis of raw data was performed using the cloud-based Maverix Biomics’ Maverix RNA-Seq analysis kit v3.0 pipeline, which utilizes a variety of well-established, open-source tools to elucidate the transcriptome and analyze differential gene expression. In brief, after preprocessing of rawsequencing reads using FastQC to remove adapter sequences, followed by filtering low-quality reads based on quality scores using PRINSEQ and Trimmomatic (42, 43), RNASeq reads are mapped to the human genome (GRCh37/hg19) using TopHat or STAR, and differentially expressed genes are identified with Cufflinks, DESeq, and/or edgeR, using a false discovery rate of 0.05 to define significantly differentially expressed genes. Analysis results are then provided through a variety of integrated graphical tools, including a private, secure version of the UCSC Genome Browser.

### Statistical analysis

Experimental results are presented as the mean plus/minus the standard error of the mean (SEM). All statistical analyses were performed using the R coding language in RStudio (RStudio, PBC, Boston, MA). One-way ANOVA was employed for multiple comparisons. A p value <0.05 was considered statistically significant. Statistical analysis of the NanoString data was performed using nSolver 4.0 (NanoString Technologies).

## Results

### Intravenous administration of purified ET3 or full-length recombinant hFVIII protein results in formation of an IgG antibody response

Purified ET3 (36, 37) or full-length recombinant hFVIII protein approved for clinical use (Kogenate® FS) were infused at a dose of 20 IU/kg for 5 weeks to pediatric animals >8 months <1 year of age (equivalent to >12 years <18 in humans) as described in the study design and depicted in **Supplementary Figure 1**. To investigate if the administration of ET3 or human FVIII protein induced anti-ET3/FVIII antibody responses, ET3/FVIII-specific IgM and IgG ELISAs were performed on plasma collected from each animal at day 0, at every week (1-5W) before protein was administered; additional analyses were also performed for ET3/FVIII-specific IgGs at weeks 7, 10, and 15.

**Figure 1:**
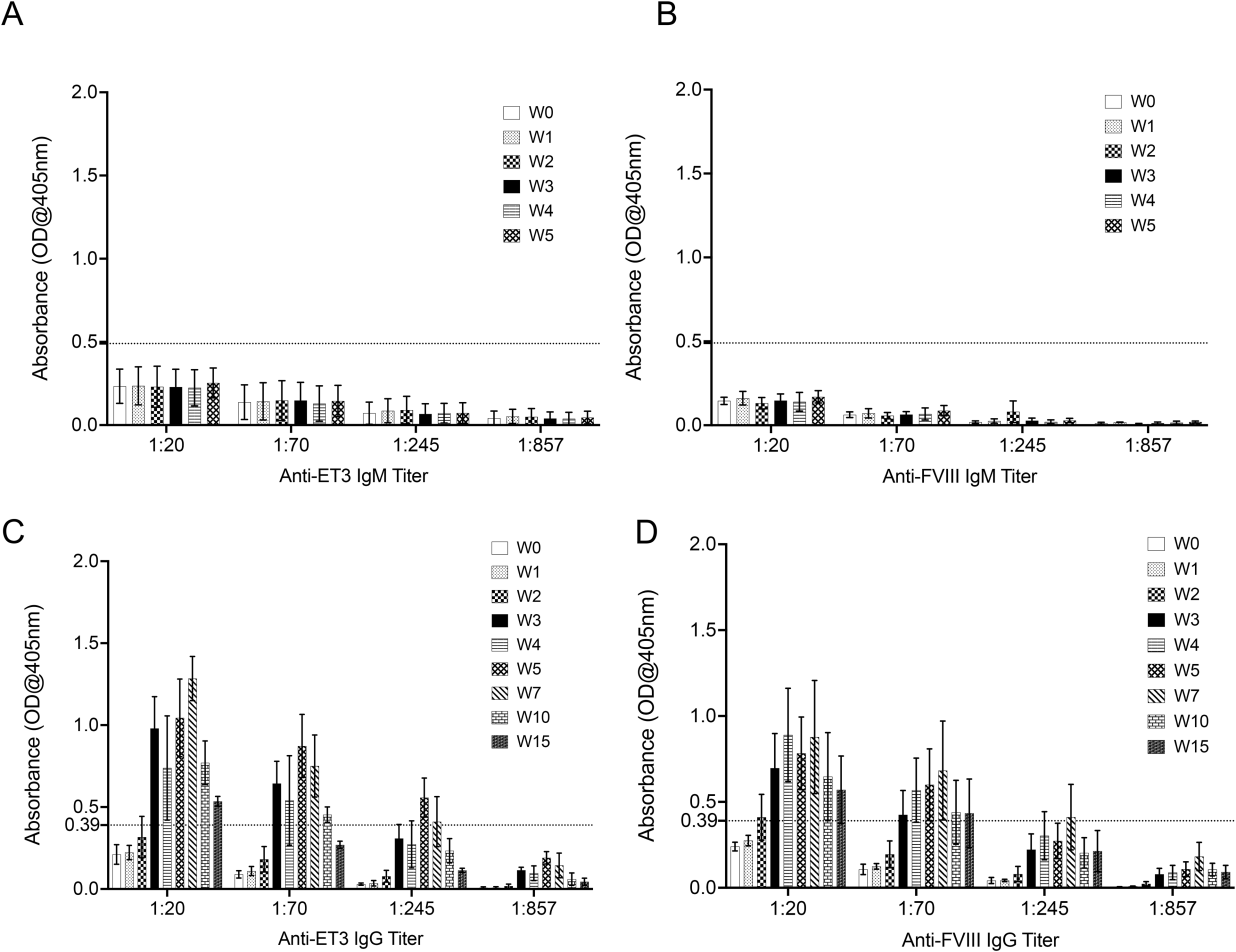
Intravenous administration of purified ET3 protein or full-length recombinant hFVIII protein results in formation of IgG antibody responses. ET3/FVIII-specific IgM and IgG ELISAs were performed on serial dilutions of plasma collected from animals that received ET3 or hFVIII protein to investigate whether ET3 and FVIII protein induced IgM and/or IgG antibodies. Positive antibody titers were defined as the dilution of plasma with an absorbance value >2 SD above the mean OD from ELISAs performed with control sheep plasma (dotted line). **(A, B)** Plasma was collected at day 0, and at every week (1-5W) before ET3 (n=3) or FVIII protein (n=3) was administered; none of the treated animals developed IgM antibodies to ET3/FVIII; **(C, D)** ET3/FVIII-specific IgG ELISAs were performed on plasma collected from each animal at day 0, at every week (1-5W) before protein was administered, and additional analyses were also performed for ET3/FVIII-specific IgGs at weeks 7, 10, and 15; **(C)** all animals that received ET3 protein developed IgG antibodies by week 3 of administration, with titers of at least 1:70. Anti-ET3 IgGs increased over time to reach titers of 1:245 in all animals at week 5. The antibody titers slowly decreased thereafter, but at week 15, titers of 1:20 were still present; **(D)** animals that received recombinant human FVIII also developed anti-FVIII IgGs antibodies starting at week 2 of administration with titers of 1:20; two out of 3 animals displayed increasing titers of IgG antibodies reaching 1:245 by week 5 and maintained this level of antibodies until at least week 7, while in one animal anti-FVIII IgGs only persisted until week 5. Data are shown as Mean± SEM.

As shown in **Figures 1A, B**, none of the animals treated with ET3 (n=3) or human FVIII protein (n=3) developed anti-ET3/FVIII IgM antibodies, which is consistent with results reported in human HA patients (44). Graphical representations of the specific anti-ET3/FVIII IgM titers present in individual animals at each time point of analysis appear in **Supplementary Figures 2A, B**, respectively. However, all animals that received ET3 protein developed IgG antibodies by week 3 of administration, with titers of at least 1:70 (**Figure 1C**). Anti-ET3 IgGs increased over time to reach titers of 1:245 in all animals at week 5 (**Figure 1C**). The antibody titers slowly decreased thereafter, but at week 15, titers of 1:20 were still present (**Figure 1C**).

Animals that received recombinant human FVIII also developed anti-FVIII IgGs antibodies starting at week 2 of administration with titers of 1:20 (**Figure 1D**). Two out of 3 animals displayed increasing titers of IgG antibodies reaching 1:245 by week 5 and maintained this level of antibodies until at least week 7 while in one animal anti-FVIII IgGs only persisted until week 5 (**Figure 1D**). Details of the specific anti-IgG titers against ET3 and FVIII protein present in individual animals at each time point of analysis appear in **Supplementary Figures 3A, B**, respectively.

### Intravenous administration of purified ET3 or full-length recombinant human FVIII protein results in formation of high-titer anti-ET3 and anti-FVIII inhibitory IgG antibodies

**Figure 2:**
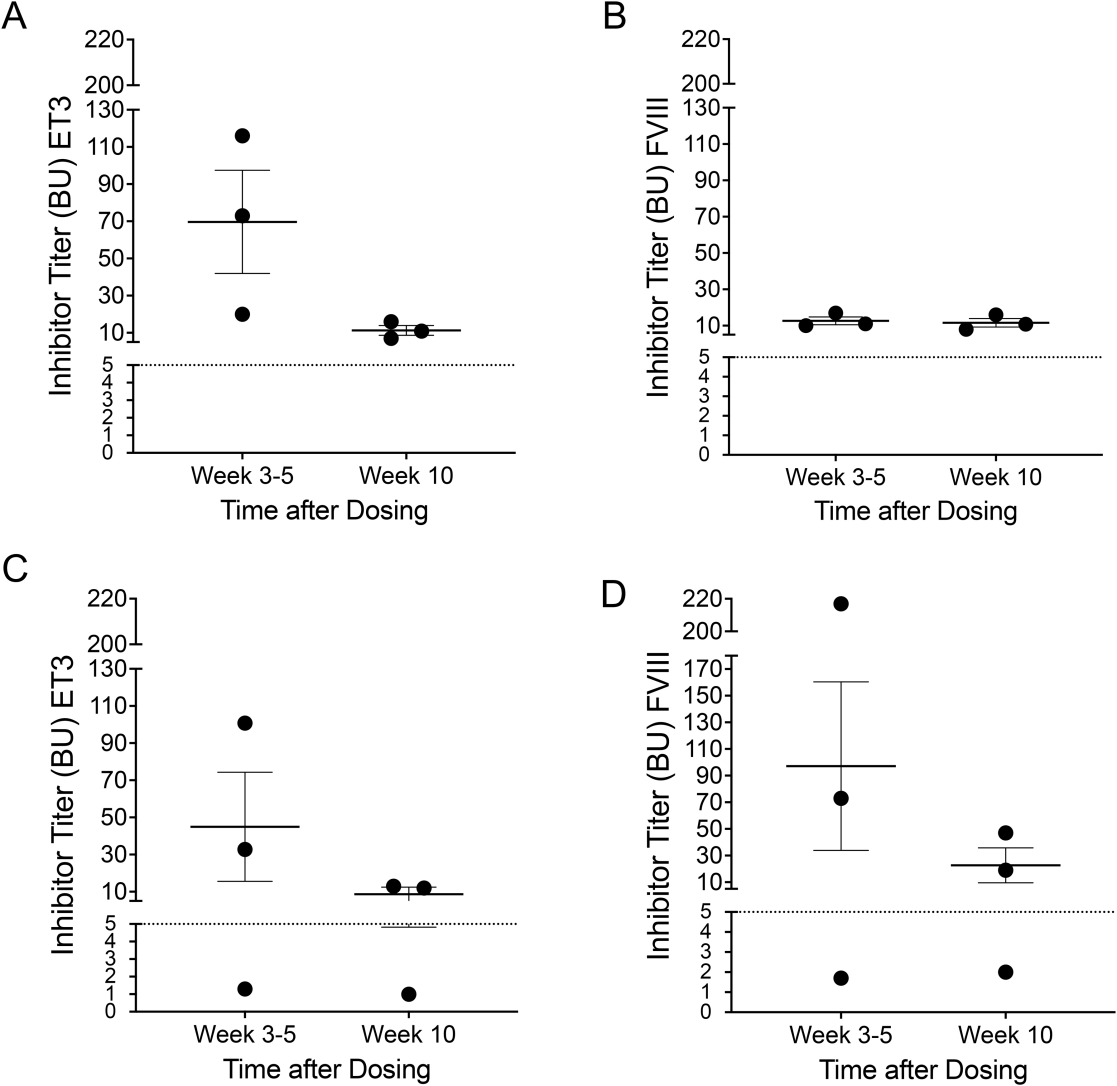
Intravenous administration of purified ET3 protein or full-length recombinant human FVIII protein results in formation of high-titer anti-ET3/FVIII IgG inhibitory antibodies. Modified Bethesda assays were performed to determine whether the anti-ET3/FVIII IgGs detected in plasma of the animals that received purified ET3 or full-length recombinant human FVIII (hFVIII) were inhibitory. **(A)** Animals that received ET3 protein displayed high-titer ET3-inhibitory antibodies with 20–116 BU/ml at week 3–5 and 7–16 BU/ml at week 10; **(B)** Cross-reactivity of ET3 antibodies with hFVIII protein with titers of 10–17 BU/ml at week 3-5, and 8–16 BU/ml at week 10; **(C, D)** Recombinant hFVIII recipients also developed inhibitory antibodies to ET3 **(C)** and hFVIII **(D)**, and they had **(D)** titers of 1.8–217 BU/ml to recombinant hFVIII by week 5, which decreased to 2–47 BU/ml by week 10 and to **(C)** ET3 protein 1.5–101 BU/ml by week 5 and 1–13 to by week 10. Data are shown as Mean± SEM.

To determine whether the anti-ET3/FVIII IgGs detected in plasma of the animals that received purified ET3 or full-length recombinant human FVIII (hFVIII) were inhibitory, modified Bethesda assays were performed. Animals that received ET3 protein displayed high-titer ET3-inhibitory antibodies with 20–116 BU/ml at week 3-5 (**Figure 2A**). The inhibitor titer in these animals then decreased to a titer of 7–16 BU/ml at week 10 (**Figure 2A**). These antibodies cross-reacted with hFVIII protein, such that the animals also had anti-hFVIII inhibitory antibody titers of 10–17 BU/ml at week 3-5, and 8–16 BU/ml at week 10 (**Figure 2B**).

Recombinant hFVIII recipients also developed inhibitory antibodies to both hFVIII and ET3 protein (**Figures 2C, D**) and had titers of 1.8–217 BU/ml to recombinant hFVIII (**Figure 2D**), and 1.5–101 BU/ml to ET3 (**Figure 2C**) by week 5. These titers decreased to 2–47 BU/ml to recombinant hFVIII (**Figure 2D**), and to 1–13 to ET3 BU/ml by week 10 (**Figure 2C**).

### Intravenous administration of PLC-mcoET3 results in increased plasma FVIII activity without the formation of anti-ET3/FVIII inhibitory antibodies

Having established that all pediatric sheep recipients, after only 3 weekly infusions of ET3 at 20 IU/kg, develop high-titer antibodies that inhibit both ET3 and hFVIII, we next investigated whether delivering ET3 protein secreted by placental cells, rather than as purified protein, would also induce the formation of ET3/FVIII inhibitory antibodies. Human PLC-mcoET3 were administered IV weekly for 3 weeks to wild-type pediatric animals (n=3) at a dose of 4x10^6^±1.3x10^6^ cells/kg, equivalent to 20 IU/kg of ET3 per infusion (cumulative dose of 60 IU/kg of ET3). No significant adverse effects were seen during or after administration of the cell therapy, in contrast to what has been reported in some human clinical trials (45). Overall, heart rates before (108±7.4) and after (118±9.1) the procedure did not change significantly (p<0.05). Of note, one of the animals displayed an elevated respiratory rate during the second infusion, which normalized within 30 minutes without any other side effects.

**Figure 3:**
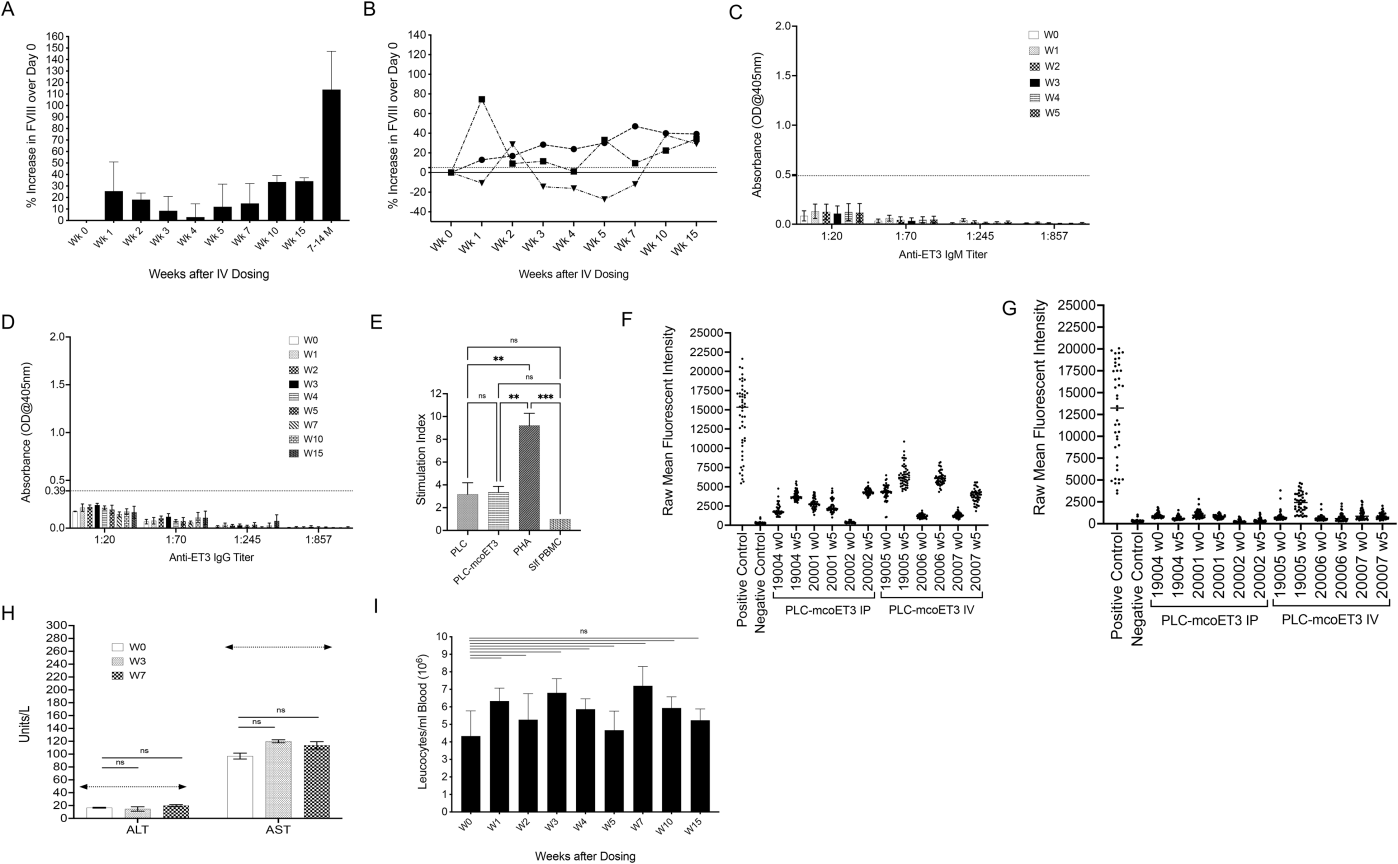
Intravenous administration of PLC-mcoET3 results in increased plasma FVIII activity without inducing anti-ET3/FVIII inhibitory antibodies, donor-specific cellular immune responses, or hepatic or hematologic alterations. Administration of PLC-mcoET3 IV weekly for 3 weeks to wild-type pediatric animals (n=3) at a dose of 4x10^6^±1.3x10^6^ cells/kg, equivalent of 20 IU/kg of ET3 per infusion, (cumulative dose of 60 IU/kg of ET3) **(A)** resulted in elevation of plasma FVIII activity, over day 0, at all-time points analyzed; **(B)** an increase in FVIII activity >5% was observed in 2 of the 3 animals at most time points; the third animal reached levels of FVIII activity comparable to the other 2 animals by W10, and these elevated levels were then maintained for another 5 weeks; **(C)** ET3-specific IgM and **(D)** ET3-specific IgG ELISAS demonstrated that none of the animals receiving PLC-mcoET3 developed anti-ET3 antibodies (positive antibody titers were defined as the dilution of plasma with an absorbance value >2 SD above the mean OD from ELISAs performed with control sheep plasma, dotted line); **(E)** one-way Mixed Lymphocyte Reactions (MLR) performed by co-culturing recipients’ peripheral blood mononuclear cells (PBMC) with same-donor PLC-mcoET3, same-donor non-transduced PLC, individual self-PBMC, and PHA as a positive control, demonstrated that the product administered was not immunogenic and did not sensitize recipients to the administered therapy; **(F, G)** solid phase pooled bead assays, using a Luminex 200 multiplex system modified for detection of antibodies in sheep serum demonstrated that the animals that received PLC-mcoET3 maintained a panel reactive antibody (PRA) status that was considered negative; displayed on the left are the animals that received PLC-mcoET3 IP, and on the right are animals that received PLC-mcoET3 IV; **(H)** Alanine aminotransferase (ALT) and Aspartate aminotransferase (AST); **(I)** white blood cell counts were analyzed at different times after birth; no statistically significant differences (ns) were found between pre-administration and after product administration at different weeks (p ≤ 0.05). Data are shown as Mean ± SEM. One-way ANOVA followed by Dunnett’s multiple comparisons tests were used to determine significant differences between day 0 and the other time points. Dotted lines denote upper normal range of AST and ALT; **p ≤ 0.01, ***p ≤ 0.001.

Plasma FVIII activity levels were determined prior to infusion, at day 0, and weekly thereafter, as described in the study design and depicted in **Supplementary Figure 1**. The percent increase in FVIII activity over day 0 was then calculated for each animal, at each respective time point, as described in the materials and methods. Overall, administration of PLC-mcoET3 IV led to elevation of plasma FVIII activity at all time points analyzed (**Figure 3A**). A >5% increase in FVIII activity at most time points was observed in 2 of the 3 animals (**Figure 3B**). The third animal, despite reaching very high levels of FVIII activity at week two, only had levels of FVIII activity comparable to the other 2 animals by W10; these elevated levels were then maintained for another 5 weeks (**Figure 3B**).

Having determined that ET3 secreted by PLC-mcoET3 was present in circulation in all 3 pediatric sheep, we next investigated whether secreted ET3 also induced an antibody response. ET3-specific IgM and IgG ELISAs were performed on plasma samples collected at day 0, and on plasma samples collected every week for 5 weeks. In addition, ET3-specific IgGs were also determined at weeks 7, 10, and 15 post-PLC-mcoET3 infusion. In similarity to the groups that received ET3 and hFVIII protein infusions, none of the animals receiving PLC-mcoET3 developed anti-ET3/FVIII IgM (**Figure 3C**). However, in stark contrast to the groups that received purified ET3 or FVIII protein, none of the recipients of PLC-mcoET3 developed anti-ET3 IgG antibodies (**Figure 3D**).

### Human PLC-mcoET3 do not induce donor-specific cellular immune responses following IV administration

Since no antibodies against ET3 were found at any time point in the animals receiving PLC-mcoET3, studies were performed to investigate whether an immune response to PLC or PLC-mcoET3 had occurred and could justify the lower levels of ET3 seen in the circulation in one of these animals. To test whether the cells administered were immunogenic and could sensitize recipients to the administered therapy, one-way Mixed Lymphocyte Reactions (MLR) were performed by co-culturing recipients’ peripheral blood mononuclear cells (PBMC) with one of the following: 1) same-donor PLC-mcoET3; 2) same-donor non-transduced PLC; 3) individual self-PBMC to establish the baseline stimulation index; and 4) Phytohemagglutinin-L (PHA) to ensure tested PBMC were not inert and could proliferate. None of the PLC-mcoET3 recipients’ PBMC displayed a significant change in proliferation when co-cultured with either transduced or non-transduced PLC, when compared to co-culture with individual self-PBMC. However, stimulation of each recipient’s PBMC with PHA resulted in significantly increased cell proliferation, demonstrating that the tested PBMC remained functional (**Figure 3E**).

Next, analyses were performed to investigate whether the human PLC-mcoET3 had induced anti-HLA Class I and/or Class II IgG antibodies. Prior to commencing these studies, we determined that the HLA Type of the PLC was: A*02,24; B*18,51; C*02,07; DRB1*04,16; DRB4*01; DRB5*02; DQB1*03(DQ7),05; DQA1*01,03; DPA1*01; DPB1*04. This knowledge was used to perform solid phase pooled bead assays, using a Luminex 200 multiplex system modified for detection of antibodies in sheep serum. Results of this screen at weeks 0 and 5 showed that none of the PLC-mcoET3 recipients that received the therapy IV had been sensitized against the donor cells, and their panel reactive antibody (PRA) status was considered negative (**Figure 3F**). Of note is that several animals displayed a higher overall raw mean fluorescent intensity (MFI) at week 5 compared to week 0. However, this increase did not correspond to the presence of antibodies specific to the donor cells; rather, it could be attributed to the presence of non-specific antibodies that were cross-reactive with the human-HLA bead targets. Assessment of anti-HLA-II antibodies in the sera these animals similarly showed that none of the animals exhibited a positive PRA, or at least a positive PRA that was specific to the transplanted cells (**Figure 3G**).

### Intravenous administration of PLC-mcoET3 does not cause hepatic or white blood cell count alterations

To evaluate whether hepatic or hematological alterations were induced because of infusing PLC-mcoET3 via the IV route, white blood cell counts (WBC), and the liver enzymes aspartate aminotransferase (AST) and alanine aminotransferase (ALT) were analyzed at W0 and at W3 and W7 after dosing. All transplanted animals maintained normal ALT and AST levels (**Figure 3H**) and had a WBC (**Figure 3I**) within normal range, that did not differ significantly from W0.

### Intraperitoneal (IP) administration of PLC-mcoET3 in pediatric sheep can increase plasma FVIII activity but induces the formation of anti-ET3/FVIII inhibitory antibodies

**Figure 4:**
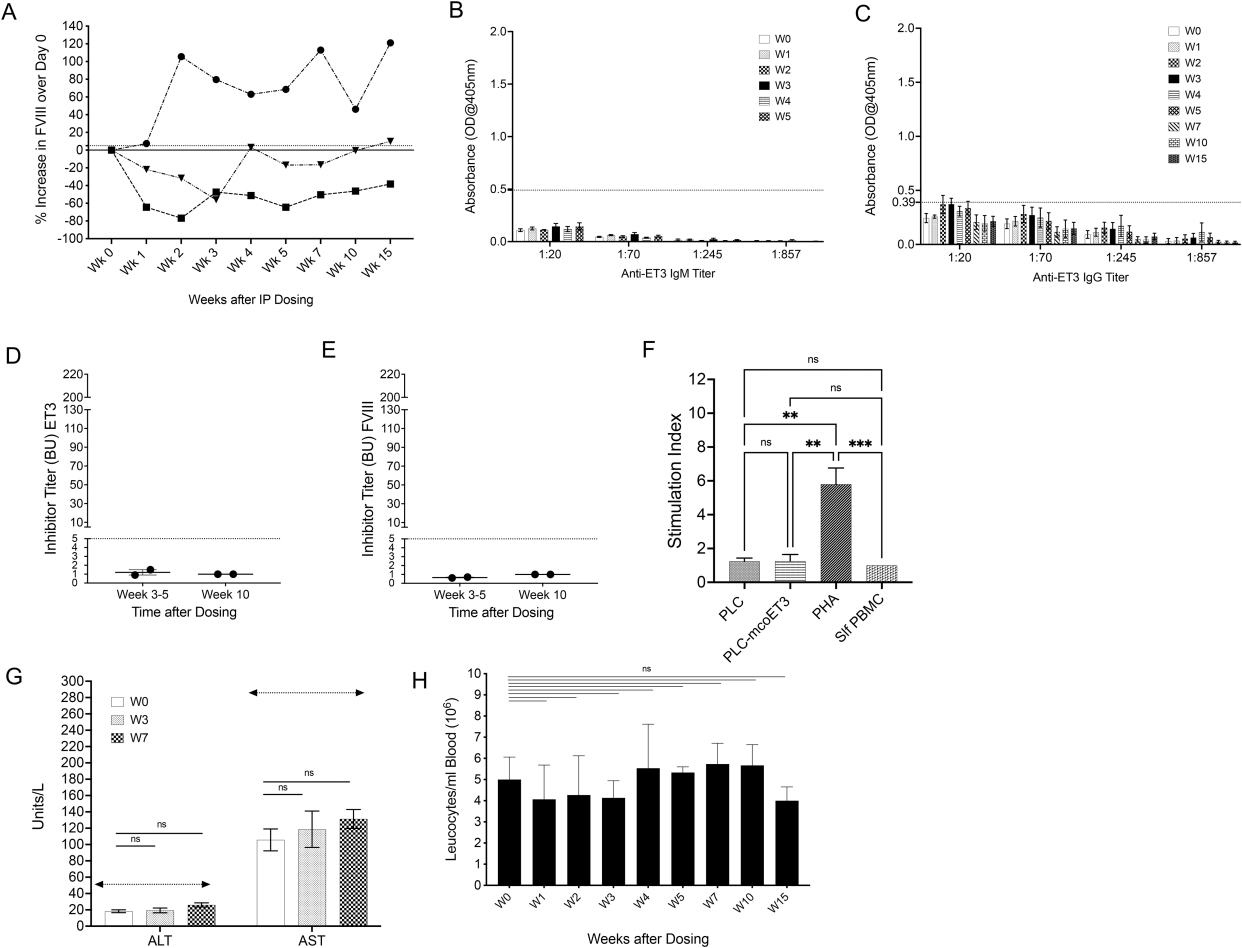
Intraperitoneal (IP) administration of PLC-mcoET3 in pediatric sheep can increase plasma FVIII activity but induces the formation of very low-titer anti-ET3/FVIII inhibitory antibodies without triggering donor-specific cellular immune responses or hepatic or hematologic alterations. Intraperitoneal (IP) delivery was also tested by administering a single bolus of PLC-mcoET3 IP (n=3) to each animal to provide a total dose of 60 IU/kg of ET3. **(A)** Only one animal had elevated levels of FVIII activity of more than 5% at week 1, and more than 20% throughout the 15 weeks of the study; **(B)** ET3-specific IgM and **(C)** IgG ELISAs were performed on serial dilutions of plasma collected at day 0, and on plasma collected every week until W5. While none of the animals developed IgM, 2 of the animals who received PLC-mcoET3 IP and had decreased FVIII activity developed very low-titer anti-ET3 IgG antibodies (positive antibody titers were defined as the dilution of plasma with an absorbance value >2 SD above the mean OD from ELISAs performed with control sheep plasma, dotted line); **(D)** modified Bethesda assays demonstrate the presence of very low-titer inhibitory antibodies at W5 and W10, against ET3 and **(E)** FVIII; **(F)** one-way Mixed Lymphocyte Reactions (MLR) performed by co-culturing recipients’ peripheral blood mononuclear cells (PBMC) with same-donor PLC-mcoET3, same-donor non-transduced PLC, individual self-PBMC, and PHA as a positive control demonstrated that the product administered was not immunogenic and did not sensitize recipients to the administered therapy; **(G)** Alanine aminotransferase (ALT) and Aspartate aminotransferase (AST); **(H)** white blood cell counts were analyzed at different times after birth; no statistically significant differences (ns) were found between pre-administration and after product administration at different weeks (p ≤ 0.05). Data are shown as Mean ± SEM. One-way ANOVA followed by Dunnett’s multiple comparisons tests were used to determine significant differences between day 0 and the other time points. Dotted lines denote upper normal range of AST and ALT; **p ≤ 0.01, ***p ≤ 0.001.

Intraperitoneal (IP) delivery was also tested as a possible way of administering PLC-mcoET3 to pediatric animals, based upon the successful outcome seen using the IP route used for HA fetal treatment (14). A single bolus of PLC-mcoET3 was administered IP (n=3) to each animal to provide a total dose of 60 IU/kg of ET3. No adverse effects were observed during or after IP administration of the cell therapy. Plasma FVIII activity levels were determined prior to administration at day 0 and weekly thereafter, as depicted in **Supplementary Figure 1**. Percentage increase in FVIII activity over day 0 for each animal was calculated as described in materials and methods. IP administration resulted in elevated levels of FVIII activity of more than 5% at week 1 and more than 20% throughout the 15 weeks of the study in only one of the pediatric sheep (**Figure 4A**), in the other 2 animals, plasma FVIII activity decreased at most time points of analysis. Since even a complete lack of engraftment of PLC-mcoET3 in the 2 animals could not justify the decrease in FVIII activity to levels lower than day 0, studies were conducted to explore the possibility that ET3 secreted by the PLC-mcoET3 administered via the IP route had induced antibodies that could inhibit FVIII activity. First, ET3-specific IgM and IgG ELISAs were performed on plasma collected at day 0, and on plasma collected every week until W5. ET3-specific IgGs were also determined weekly and at weeks 7, 10, and 15 post-IP PLC-mcoET3 administration. In similarity to all the previous groups, none of the animals in this study developed IgM (**Figure 4B**), but in contrast to the group that received PLC-mcoET3 via the IV route, the 2 animals who received PLC-mcoET3 IP and had decreased FVIII activity developed low-titer anti-ET3 IgG antibodies (**Figure 4C**). However, the animal that exhibited significantly elevated levels of FVIII activity throughout the study did not develop anti-ET3 IgG antibodies at any time point of evaluation. It is important to note that the anti-ET3 IgG antibodies developed after PLC-mcoET3 IP administration were lower in titer and of shorter duration than those seen in animals that received purified ET3 protein. Details of the specific anti-IgG titers against ET3 protein present in individual animals at each time point of analysis appear in **Supplementary Figure 4**. Specifically, one animal had IgG antibodies with a titer of 1:70 at W2, the titer decreased to 1:20 at W3 and were undetectable at W4. The other animal had borderline detectable IgG antibodies at W5, with a titer of 1:20.

To determine whether the anti-ET3/FVIII IgGs detected were inhibitory, modified Bethesda assays were also performed. In contrast to what was seen in animals that received purified hFVIII or ET3 protein, only very low-titer inhibitory antibodies of 1 BU to ET3 at W5 and W10 were detected (**Figure 4D**). These very low-titer inhibitory antibodies also cross-reacted with hFVIII, with a titer of 1 BU to hFVIII seen at W10 (**Figure 4E**).

### Human PLC-mcoET3 do not induce donor-specific cellular immune responses when administered IP

To test whether the PLC-mcoET3, when administered IP, induced an immune response sensitizing recipients to the therapy, one-way MLR were performed, co-culturing each recipient’s PBMC with identical stimuli and controls as described above. None of the PLC-mcoET3 IP recipient’s PBMC exhibited a significant change in proliferation when co-cultured with either transduced or non-transduced PLC, when compared to co-culture with individual self-PBMC, while stimulation of each recipient’s PBMC with PHA resulted in significantly increased cell proliferation, demonstrating that the tested PBMC remained functional (**Figure 4F**).

In addition, solid phase pooled bead assays were performed as described above to study whether the IP administration of human PLC-mcoET3 had induced anti-HLA Class I and/or Class II IgG antibodies. Results of this screen at weeks 0 and 5 showed that none of the PLC-mcoET3 recipients that received the therapy IP had been sensitized against the donor cells, and each animal’s PRA was considered negative (**Figure 3F**). Likewise, assessment of anti-HLA-II antibodies in the sera these animals showed that none of the animals exhibited a positive PRA, or at least a positive PRA that was specific to the transplanted cells (**Figure 3G**).

### IP administration of PLC-mcoET3 does not cause hepatic or WBC count alterations

To evaluate whether hepatic or hematological alterations were induced by the IP administration of PLC-mcoET3, WBC and the liver enzymes AST and ALT were analyzed at W0 and at W3 and W7 after dosing. All transplanted animals maintained normal ALT and AST levels (**Figure 4G**) and had a WBC (**Figure 4H**) within normal range, that did not differ significantly from W0.

### ET3-specific memory T_h1_ and T_h2_ lymphocytes are not present in any of the ET3/FVIII protein or PLC-mcoET3 IP recipients

**Figure 5:**
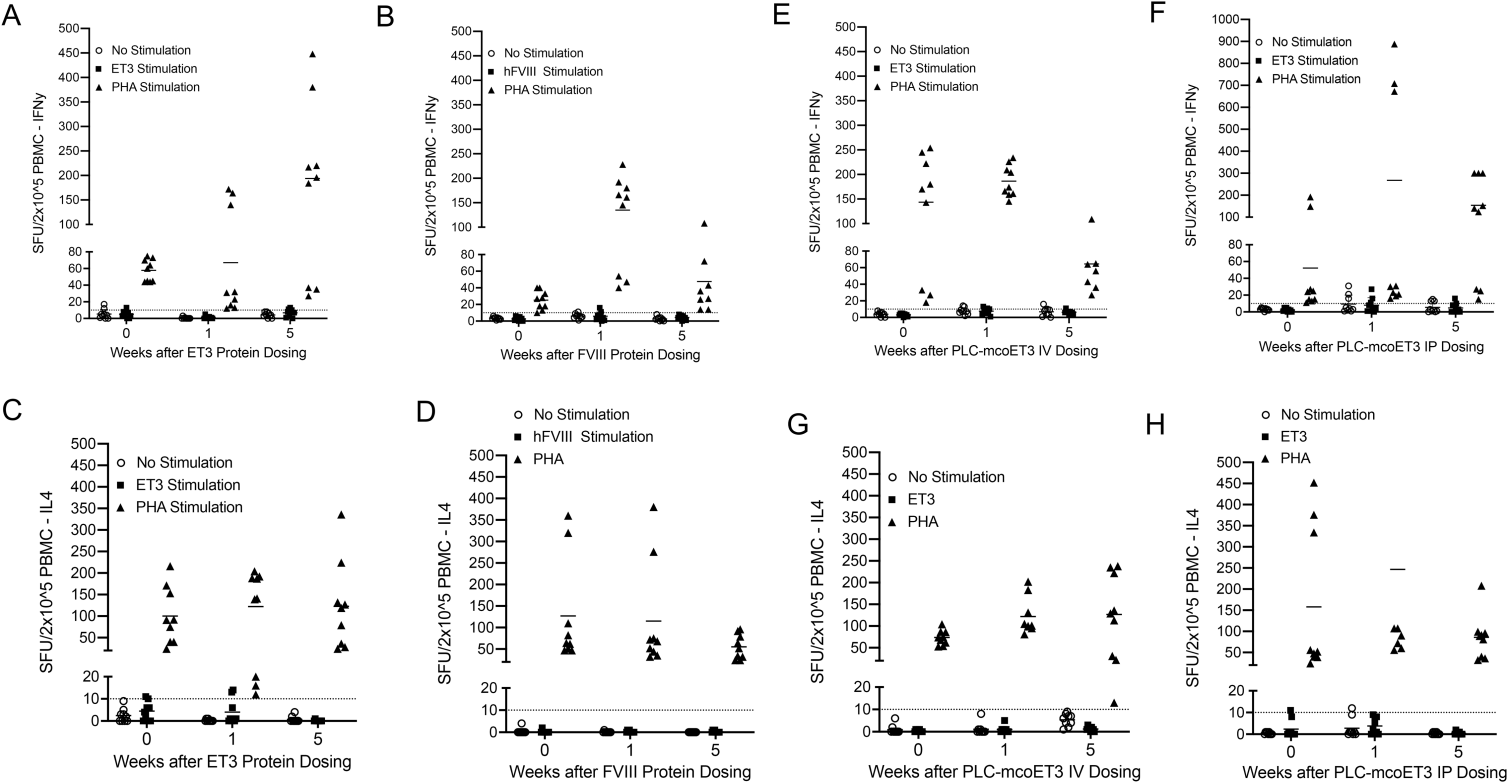
ET3-specific memory T_h1_ and T_h2_ lymphocytes are not detected in any of the ET3/FVIII protein or PLC-mcoET3 recipients. **(A, B)** IFN-γ and **(C, D)** IL-4 ELISpot assays were performed to determine the presence of reactive ET3/FVIII-specific memory T cells in sheep before administration of ET3 and hFVIII protein, and at W1 and W5. **(E, F)** IFN-γ and **(G, H)** IL-4 ELISpot assays were performed to determine the presence of reactive ET3/FVIII-specific memory T cells in sheep before administration of the cell product IV or IP, respectively, and at W1 and W5 post-dosing. A positive response was defined by a Stimulation Index >2, and > 10 SFU/2x10^5^ PBMC (dotted line). ET3-specific T_h1_ or T_h2_ lymphocytes were not detected by ELISpot in any of the animals that developed ET3/FVIII antibodies against the respective purified ET3 or hFVIII proteins nor the sheep that received PLC-mcoET3 IV or IP.

To determine whether ET3-specific memory T cells could be detected in those pediatric sheep who developed ET3/FVIII specific IgGs, IFN-γ and IL-4 ELISpot assays were performed before administration of the product(s) and at W1 and W5 to determine the presence, secretion intensity, and frequencies of reactive ET3-specific memory T cells. A response was considered positive if the Stimulation Index (SI) was >2, and if SFU/2x10^5^ PBMC >10. Unexpectedly, given the robust anti-FVIII/ET3 humoral immunity seen in the animals that received purified FVIII/ET3 protein, ET3-specific T_h1_ or T_h2_ lymphocytes were not detected by ELISpot in any of the animals that developed ET3/FVIII antibodies against the respective purified ET3 or FVIII proteins (**Figures 5A–D**) nor the sheep that received PLC-mcoET3 IV or IP (**Figures 5E–H**). Importantly, all animals’ lymphocytes maintained the ability to react and secrete IFN-γ and IL-4 when stimulated with PHA, demonstrating that their lymphocytes were functional, able to be activated, and capable of secreting the assayed cytokines. An important caveat to these studies is that our inability to detect memory T cells in the animals exhibiting high-titer inhibitors could be due, at least in part, to possible difficulty of detecting these cells in peripheral blood (as few may circulate) and/or technical challenges of the assays, rather than an actual lack of memory, which would be hard to reconcile with the robust antibody response.

### Immune cell gene expression signatures are different in animals that received PLC-mcoET3 from those who received ET3/FVIII protein

**Figure 6:**
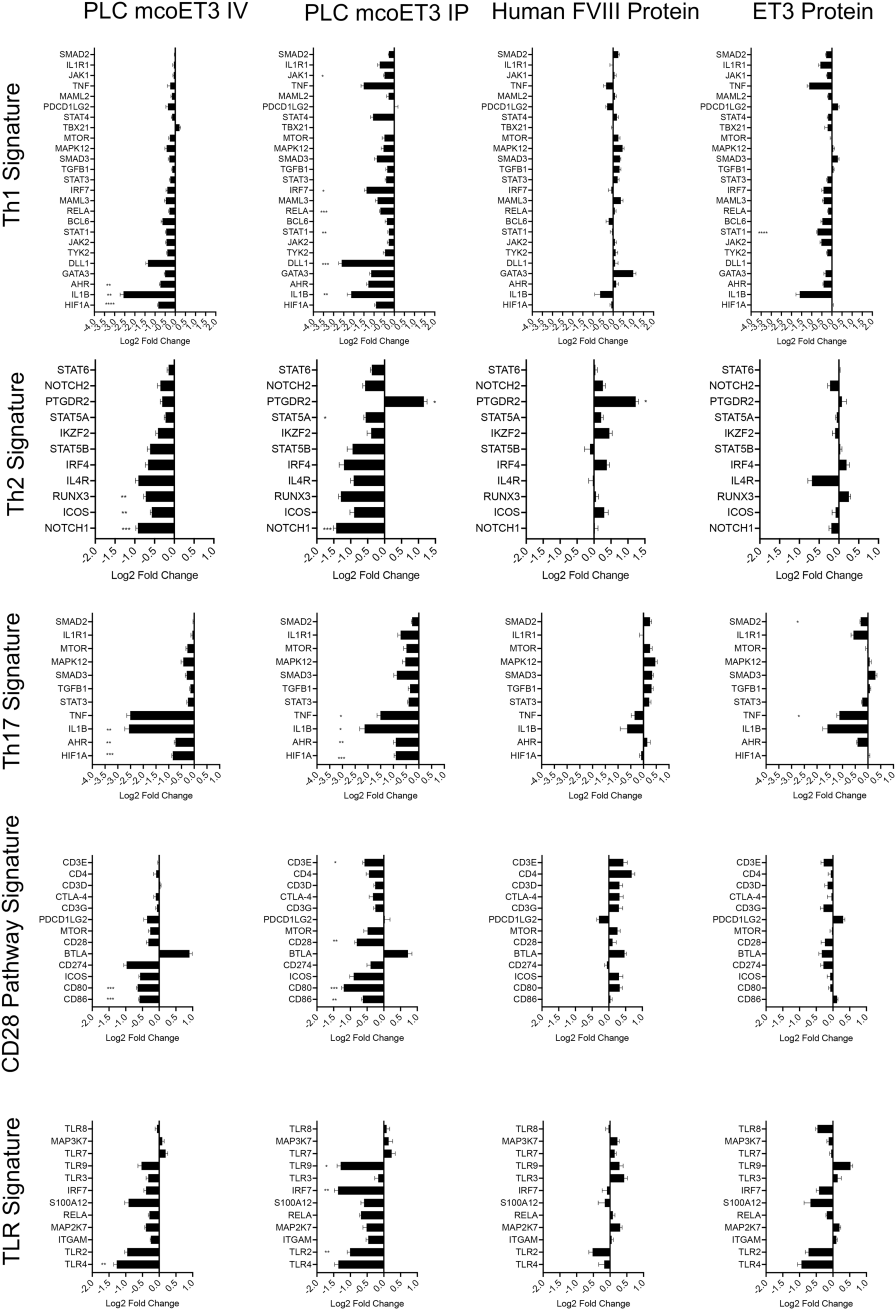
Immune cell gene expression signatures are different in animals that received PLC-mcoET3 from those that received ET3/FVIII protein. NanoString custom-designed ovine-specific 165-target multiplex gene expression was performed on total mRNA isolated from PBMC collected from each of the animals at W0 as a baseline, and at W5 after product administration. Th1, Th2, and Th17 responses, antigen presentation, toll-like receptor (TLR) cascades, and co-stimulation via CD28 family members signature transcripts can be seen in IV and IP recipients of PLC-mcoET3 and in sheep who received hFVIII and ET3 proteins. *p ≤ 0.05, **p ≤ 0.01, ***p ≤ 0.001.

Because clear Th1 or Th2 responses were not detected in sheep that developed anti-FVIII/ET3 antibodies, and to gain a mechanistic understanding of how IV infusion of ET3/FVIII protein, and administration of PLC-mcoET3 IP or IV, altered immune response, a NanoString custom-designed ovine-specific 165-target multiplex gene expression assay (**Supplementary Table 1**) was performed on total mRNA isolated from PBMC collected from each of the animals at W0 as a baseline, and at W5 after product administration. This assay encompassed genes involved in T cell signaling, Th1, Th2, and Th17 responses, antigen presentation, toll-like receptor (TLR) cascades, and co-stimulation via CD28 family members. Recipients of PLC-mcoET3 IV displayed downregulation of Th1, Th2, and Th17 signature transcripts and downregulation of most transcripts associated with TLR signaling and components of the CD28 family of receptors involved in the activation of naïve T cells (**Figure 6**). By contrast, sheep who received FVIII protein showed overall upregulation of transcripts involved in T cell immune responses, while surprisingly, ET3 recipients only exhibited upregulation of a few transcripts, none of which, with the exception of TLR9, are known to be involved in the development of FVIII inhibitors (46) (**Figure 6**, **Supplementary Figure 5**).

### Exosomes secreted by mcoET3-PLC contain multiple mRNA transcripts that can contribute to blunting of anti-FVIII immunity

As MSC can exert their biological effects via transfer of exosomes, and to investigate whether exosomes’ mRNA content could contribute to the absence of FVIII immune response in animals that received ET3 protein produced by the infused cells, RNAseq was performed on exosomes isolated from mcoET3-PLC supernatants, and analyses were performed focusing specifically on transcripts for proteins that have been implicated in tolerance to FVIII. These analyses revealed the presence of multiple transcripts within the mcoET3-PLC exosomes that could have played a role in the observed blunting of anti-FVIII immunity (**Table 1**).

**Table 1:** mRNAs present in exosomes of mcoET3-PLC that have been implicated in immune tolerance to FVIII.

Gene ID	Gene name	Function
HMOX1	Heme oxygenase 1	HMOX1 has been shown to be key in induction of T-regs and in tolerance to FVIII and lower incidence of FVIII inhibitors in humans
IDO1	Indoleamine 2,3-dioxygenase 1	Participates in the induction of immune regulatory pathways in various immune cells including T cells and dendritic cells,preventing inhibitors to FVIII, perhaps through TLR9
TGF-b	Transforming Growth Factor b	Can help to convert conventional T cells into Tregs
CTLA-4	Cytotoxic T-lymphocyte associated protein 4	Can block T cell activation. Tregs upregulate CTLA-4 to promote tolerance
PDCD1	Programmed Cell Death 1	Found on FVIII-specific B cells, this molecule, when activated by its ligand PD-L1, triggers apoptosis, thereby removing inhibitor-forming B cells. The PD-1 pathway is also known to induce Tregs
CD274/PDL-1	Programmed Cell Death Ligand 1	Expressed on FVIII-specific Tregs, PD-L1 ligates with PD-1 on B cells, inducing B cell apoptosis during successful ITI
HLA-E	Major Histocompatibility Complex, Class I, E	A non-classical class I MHC protein involved in Immunomodulation/tolerance
HLA-F	Major Histocompatibility Complex, Class I, F	A non-classical class I MHC protein involved in Immunomodulation/tolerance
HLA-G	Major Histocompatibility Complex, Class I, G	A non-classical class I MHC protein involved in Immunomodulation/tolerance
AIRE	Autoimmune Regulator	Although normally associated with central tolerance, deletional tolerance has also been shown to be mediated by extrathymic Aire-expressing cells
FLT3L	Fms related receptor tyrosine kinase 3 ligand	FLT3L selectively expands plasmacytoid DCs (pDCs), resulting in increased Treg induction
IL-27	Interleukin-27	IL-27 promotes the development of Tregs and tolerogenic dendritic cells (tolDCs)
IL-10	Interleukin-10	IL-10 also promotes the development of Tregs and tolDCs

### PLC-mcoET3 can be detected in tissues of pediatric sheep after IP and IV administration at 7–18 months post-administration and upregulate several tissue cell-specific transcripts

**Figure 7:**
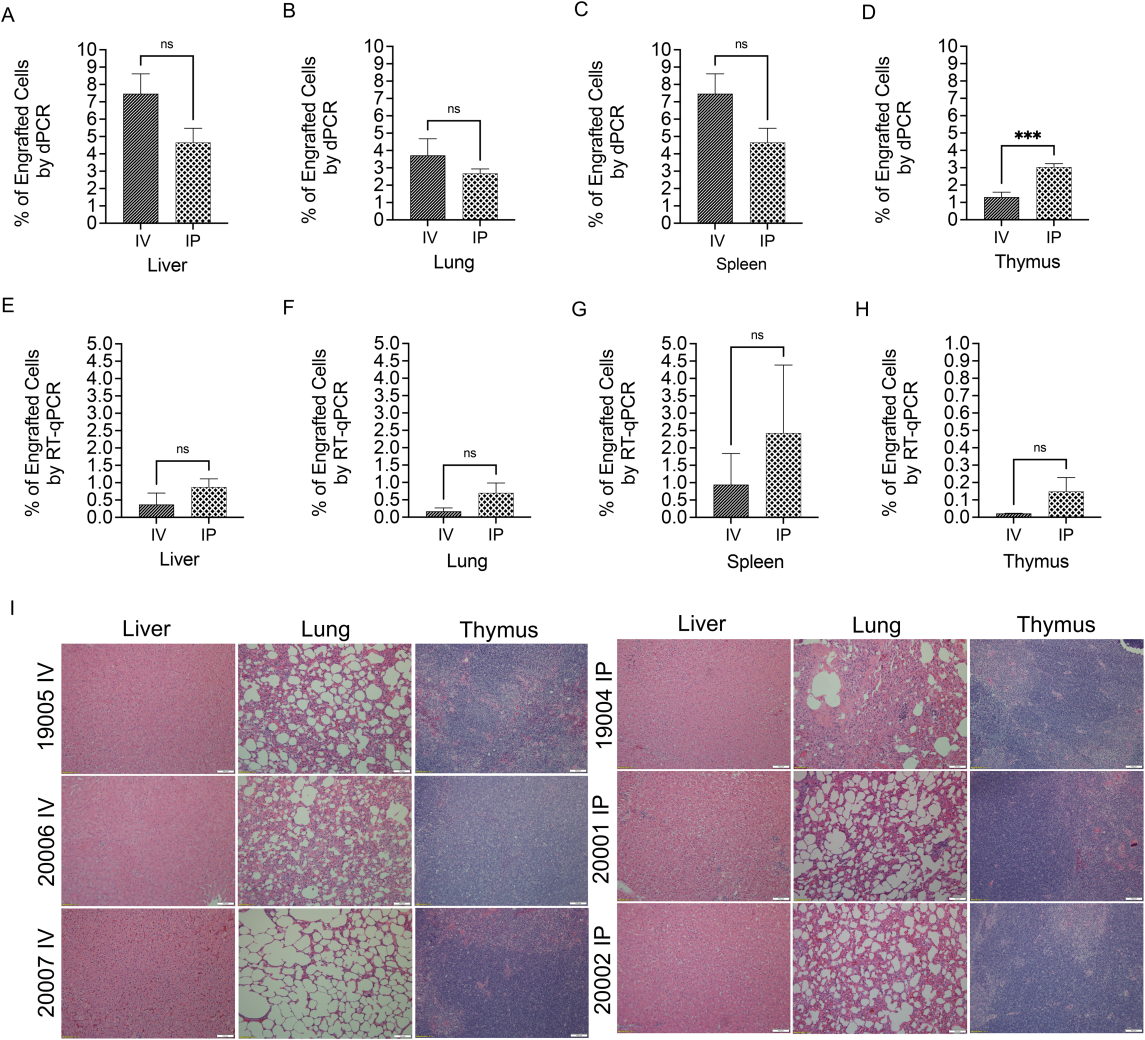
PLC-mcoET3 can be detected in tissues of pediatric sheep after IP and IV administration at 7–18 months post-administration by dPCR and by RT-qPCR. dPCR using human Alu-specific primers was performed in triplicate on DNA extracted from liver, lung, spleen, and thymus. All the tissues analyzed contained human DNA; the same tissue samples from control non-treated animals showed no target amplification. RT-qPCR was performed in triplicate using primers specific for mcoET3 and sheep GAPDH. Percentage of engraftment was determined by comparing ΔCt values to a standard curve consisting of increasing percentages of mcoET3-PLC (0, 0.01, 0.1, 1, 5, and 10%) in sheep stromal cells. (AH) Percentages of human cells present in liver **(A, E)**, lung **(B, F)**, spleen **(C, G)**, and thymus **(D, H)** as determined dPCR **(A–D)** and RT-qPCR **(E–H)** using human Alu- and mcoET3-specific primers, respectively; **(I)** H&E staining was also performed on sections from each tissue to determine whether engraftment of PLC-mcoET3 in each of the different tissues caused any untoward effects. Slides of the tissue sections were sent to a certified animal pathologist. Results showed no evidence of any lentivector-related toxicity in any of the tissues examined. Moreover, there were no changes in tissue architecture, ectopic tissue formation, gross tumor development, or areas of atypical cells or foci of hyperplasia or neoplasia. All images were acquired with a Leica DM4000 B microscope using a 10x objective. *p ≤ 0.05, **p ≤ 0.01, ***p ≤ 0.001, ns, not significant.

Next, studies were performed to determine whether the IV- and IP-transplanted PLC-mcoET3 persisted long-term in the various tissues and, if so, to define the sites of lodging and the relative percentage of PLC-mcoET3 within the parenchyma of each engrafted organ. Recipients were euthanized at 7–18 months post-PLC-mcoET3 administration, DNA and RNA were isolated from samples of liver, lung, spleen, and thymus, and dPCR and RT-qPCR were performed, using human Alu- and mcoET3-specific primers. Percentages of human cells present in liver, lung, spleen, and thymus (**Figures 7A–D**) as determined by Alu-specific dPCR showed no significant differences between the IV and IP groups, with the exception of thymus, in which IP injection led to higher numbers of engrafted cells. Quantification of human cells in sheep tissues based on expression of the transgene at the RNA level (**Figures 7E–H**) led to the extrapolation of much lower levels of human cells than those determined by dPCR, but in similarity, statistically significant differences in percentages of human cells were not found between the 2 cell-treated groups. Since dPCR has roughly 10-fold greater sensitivity than RT-qPCR, it is possible that the discrepancies in the results between the 2 different methods reflect the difference in sensitivity of the methods employed. It is also possible that samples that were harvested for DNA and RNA extraction contained different percentages of human-derived cells. To determine whether PLC-mcoET3’s distribution in tissues was homogeneous, tissue sections were examined by immunohistochemistry using an antibody specific to the human nuclear protein Ku80. This analysis showed that while regions containing PLC-mcoET3 can be seen in localized areas within the liver and lung parenchyma, many other areas of these same tissues are completely devoid of human (Ku80-positive) cells. Since organ size in adult sheep makes it impossible to analyze the entire organ, it is possible that even with multiple organ sampling, the levels of human cells can be overestimated or underestimated depending upon the sites of collection (**Supplementary Figure 6**).

To investigate whether PLC-mcoET3 that lodge in different tissues assumed transcriptomes identical to those of organ-specific cell types, RNA was isolated from liver, lung, and thymus, and transcriptome RNA-seq libraries were created and aligned to *Homo sapiens* libraries, after eliminating any possible cross-reactivity with ovine transcripts. Transcriptome RNA-seq libraries were also prepared from PLC-mcoET3 prior to transplant. PLC-mcoET3 transcripts were then compared with the human transcripts known to be expressed in tissue-specific cells, and with the human transcripts present in the organs of the transplanted animals. The results depicting the human liver-, lung- and thymus-specific upregulated transcripts are shown in **Supplementary Tables 2–4**. Although cell-specific transcripts were found to be upregulated in the PLC-mcoET3 that lodged in specific tissues, a whole transcriptome profile consistent with that of a particular fully differentiated tissue-specific cell type was not present. These results also corroborate the results above demonstrating that PLC-mcoET3 remain in tissues long-term.

### Histopathological evaluation of tissues from PLC-mcoET3 transplanted animals

H&E staining was also performed on sections from each tissue to determine whether engraftment of PLC-mcoET3 in each of the different tissues caused any untoward effects. Slides of the tissue sections were sent to a certified animal pathologist. Results showed no evidence of any lentiviral-related toxicity in any of the tissues examined. Moreover, there were no changes in tissue architecture, ectopic tissue formation, gross tumor development, or areas of atypical cells or foci of hyperplasia or neoplasia. Representative images of these tissue sections can be seen in **Figure 7I**.

### Treatment of animals with pre-existing inhibitors with PLC-mcoET3

**Figure 8:**
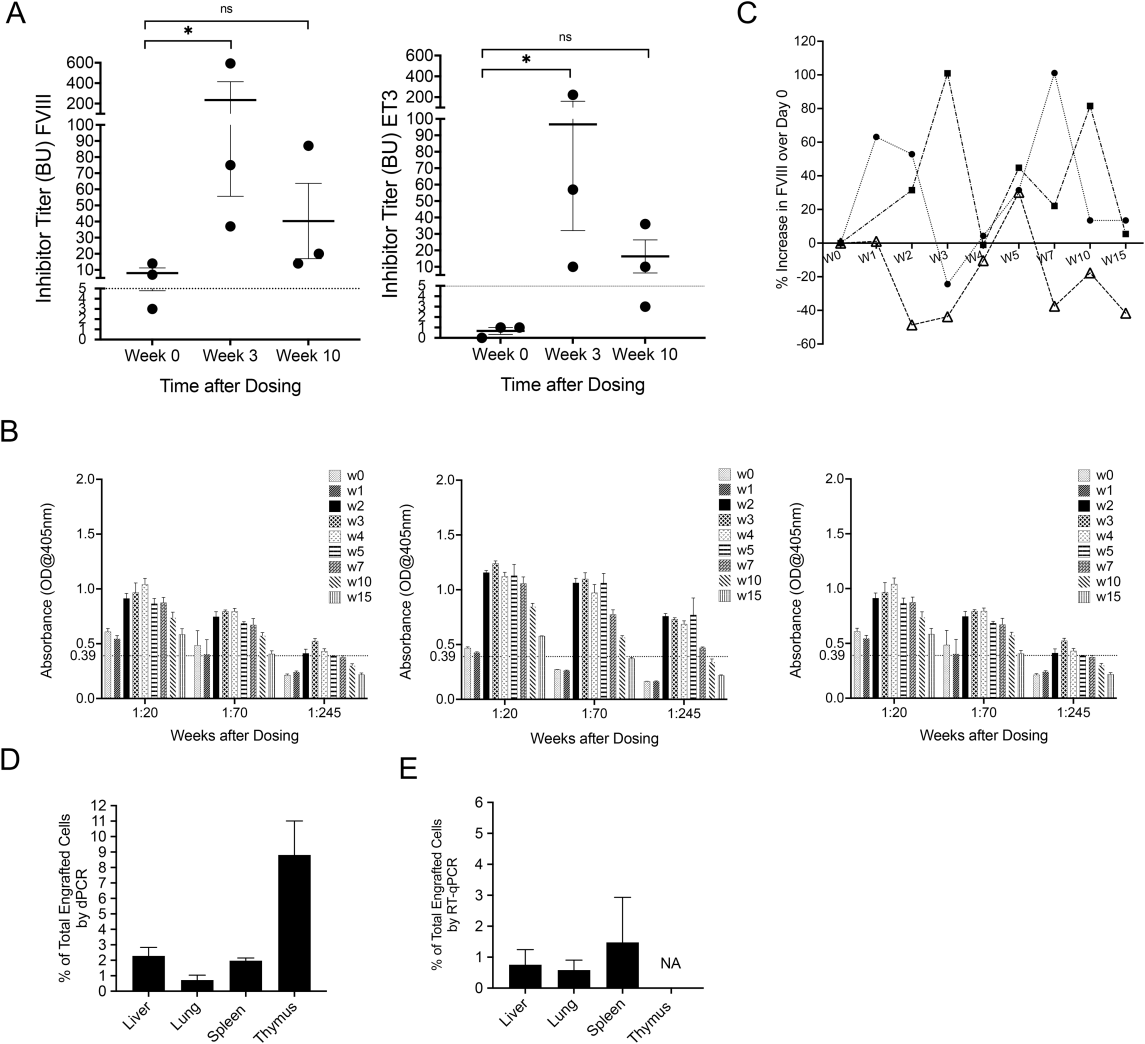
Treatment of animals with pre-existing inhibitors with PLC-mcoET3 results in an increase in FVIII activity in two of three animals at most of the time points analyzed despite the rise in titers of inhibitory antibodies. Human PLC-mcoET3, producing 3.6 IU/10^6^cells/24hr of FVIII/ET3, were administered to 3 animals on 3 consecutive weeks (20IU/kg/24h) to reach a total dose of 60IU/kg/24h at 14–23 months after the administration of ET3 and/or FVIII protein. **(A)** Animals were chosen because they continued to harbor FVIII and ET3 inhibitors at the time of PLC-mcoET3 infusion; **(B)** PLC-mcoET3 infusion triggered an increase in inhibitors against ET3 (p<0.05) and FVIII (p<0.05) at W3, when compared to W0 in all 3 animals; **(C)** FVIII activity increased in two out of three animals at most of the time points analyzed, despite the rise in titers of inhibitory antibodies; **(D)** dPCR and **(E)** RT-qPCR were performed as described above, and PLC-mcoET3 were found to be present in all tissues analyzed. *p<0.05, **p<0.01, ***p<0.001. ns, not significant.

Given the increase of FVIII activity in plasma and absence of an immune response to ET3 in animals that received PLC-mcoET3 via the IV route, studies were performed to determine whether IV administration of PLC-mcoET3 could overcome pre-existing FVIII/ET3 inhibitors in those animals that had received ET3 and hFVIII protein. Human PLC-mcoET3, producing 3.6 IU/10^6^cells/24hr of FVIII/ET3, were administered to 3 animals in 3 consecutive weeks (20IU/kg/24h) to reach a total dose of 60IU/kg/24h dose at 14–23 months after the administration of ET3 and/or hFVIII protein. These animals were chosen because they continued to harbor hFVIII and ET3 inhibitors (**Figure 8A**) at the time of PLC-mcoET3 infusion. PLC-mcoET3 infusion, triggered an increase in FVIII/ET3 inhibitors against ET3 (p<0.05) and hFVIII (p<0.05) at W3, when compared to W0 in all 3 animals (**Figure 8B**), with anti-ET3 IgGs peaking at W3-W4 and persisting until W15 (**Figure 8B**). Interesting is that plasma FVIII activity increased in two out of three animals at most of the time points analyzed (**Figure 8C**), despite the rise in titers of inhibitory antibodies. The third animal exhibited a significant decrease to below baseline levels (W0) of FVIII activity starting at W2 post-PLC-mcoET3 infusion (**Figure 8C**).

To investigate whether the presence of inhibitory antibodies at the time of PLC-mcoET3 infusion, and increase in the subsequent weeks, resulted in rejection of the cell product, the presence of human cells in different tissues was quantified by dPCR and RT-qPCR as described above, and PLC-mcoET3 were found to be present in all tissues analyzed (**Figures 8D, E**).

To examine whether the infused PLC-mcoET3 present in different tissues assumed transcriptomes identical to those of organ-specific cell types, we performed the same experiments detailed above, and the results are shown in **Supplementary Tables 2–4**. Once again, although cell-specific transcripts were found to be upregulated in the PLC-mcoET3 that lodged in specific tissues, a whole transcriptome profile consistent with that of a particular fully differentiated tissue-specific cell type was not present.

## Discussion

These studies tested the ability of human placenta-derived stromal cells (PLC) to deliver a codon-optimized, bioengineered human/porcine hybrid FVIII (ET3) (11–13) and thereby increase plasma FVIII levels, and investigated the immunological consequences of this approach to FVIII delivery. Importantly, data showed that the IV bolus infusion of ET3 protein or purified hFVIII led to a robust humoral immune response, with all animals forming high-titer IgG antibodies that were inhibitory and cross-reacted with both hFVIII and ET3. In similarity to human HA patients who develop inhibitors, these animals formed high-titer IgG inhibitors without generating a prior IgM response. In stark contrast to the results seen in animals receiving purified hFVIII or ET3 protein, IV infusion of PLC-mcoET3 led to long-lasting elevation in plasma FVIII activity levels without inducing high-titer antibodies, thus demonstrating that the effect on the immune response is markedly different when PLC are used to deliver the same protein. Therefore, it was unexpected that 2 out of 3 animals that received PLC-mcoET3 through a different administration route, IP, also developed ET3-specific antibodies. It is important to note, however, that the antibodies that did form in these IP-treated animals were very low-titer, of short duration, and they did not occur in all animals in this treatment group. Indeed, the IP PLC-mcoET3-treated animal that did not form ET3-specific antibodies exhibited the highest plasma FVIII activity levels of any animal in the present report, and these elevated levels persisted for the duration of this 15-week study. Notably, neither the IV nor IP infusion of PLC-mcoET3 led to an immune response specific to the human PLC, nor did it trigger any hematologic or hepatic alterations.

While some prior studies have suggested that stromal cells do not have the ability to engraft in postnatal animals (or human patients) [*reviewed in* (47)], our data provide compelling evidence that PLC-mcoET3 lodge in multiple tissues following IV or IP transplantation into pediatric sheep, and that these cells were able to persist for at least 18 months (the latest time point at which an animal was euthanized for analysis). As the transplanted PLC-mcoET3 likely take time to traffic to and lodge within the various tissues, it is not surprising that the plasma levels of FVIII in many of the PLC-mcoET3 recipients were initially low and then steadily increased to level off once the transplanted PLC-mcoET3 had stably located within various tissues. Future longer-term studies would be needed to determine the duration that engrafted cells persist in these tissues and define how FVIII levels change over time after treatment to ascertain if and when additional PLC-mcoET3 administration would be necessary to maintain therapeutic effect. An important caveat to using PCR-based analysis to quantitate engraftment in the various tissues is that, although samples from different parts of each tissue’s parenchyma were harvested for DNA and RNA extraction, it is possible that the levels of engraftment obtained by PCR may not reflect the overall level of engraftment in the whole organ, due to the likely non-homogeneous distribution of PLC throughout the tissue.

Interestingly, no ET3-specific Th1 or Th2 cells were detected (by ELISpot) in any of the animals that received either purified ET3 protein or PLC-mcoET3, suggesting that the inhibitory antibodies generated were neither Th1- nor Th2-dependent. Immune cell signaling gene expression signatures obtained from animals’ PBMC obtained using NanoString technology provided information regarding the differential expression of key genes driving the response of the recipients’ immune systems following IV infusion of hFVIII and ET3 proteins versus receiving ET3 through the transplantation of PLC-mcoET3 via either the IV or IP route.

In the PBMC from animals that received PLC-mcoET3 IV, transcripts for hypoxia-inducible factor 1 (HIF1α), aryl-hydrocarbon receptor (AHR), CD80, CD86, and toll-like receptor 4 (TLR4) were all significantly downregulated at W5 when compared to W0. HIF1α has been shown to be required for dendritic cell (DC) maturation and migration and Th17 differentiation, and to negatively regulate the differentiation of Tregs (48). AHR is required for optimal B‐cell proliferation.

GATA3, RUNX3, and mTOR transcripts were also downregulated in this group of animals at W5, however the decrease was not found to be statistically significant due to intergroup variability. GATA3 and RUNX3 both play key roles in regulating T cell development, proliferation, and differentiation, including the transition to memory T cells (49, 50), and mTOR is a critical regulator of immune function that senses and integrates cues from the immune microenvironment, thereby promoting differentiation, activation, and function of T cells, B cells, and antigen-presenting cells (APC) (51, 52). Its vital role in multiple aspects of the immune response led Herzog and colleagues to devote many years to exploring and validating the use of various approaches to inhibit mTOR during FVIII administration as a means of inducing tolerance to FVIII (9, 53–56). Therefore, downregulation of any one of these transcripts following PLC-mcoET3 administration could impact multiple pathways that could help to promote tolerance, rather than immunity, to ET3.

The administration of PLC-mcoET3 also led to the direct downregulation of the transcript for the co-stimulatory molecule ICOS and of the ICOS-regulators IKZF2 and NFATC1. As transient blockade of ICOS at the time of FVIII administration has been shown by Miao and colleagues to generate long-term tolerance to FVIII (57), the downregulation of both ICOS itself and two other TFs that regulate its expression thus provides another very plausible mechanism by which the delivery of ET3 by PLC enabled evasion of an immune response.

The avoidance of so-called “danger signals” (58, 59) at the time of FVIII administration has long been thought to be critical to avoiding the induction of inhibitors (60–63). A key family of molecules that sense such danger signals and activate the appropriate immune signaling pathways to protect the host from what are perceived to be foreign entities is the toll-like receptor (TLR) family (64, 65). Indeed, several studies have provided compelling evidence that the triggering of TLR signaling at the time of FVIII administration or while performing immune tolerance induction (ITI) leads to the formation of inhibitors or the failure to achieve tolerance, respectively (66–68). The observed pronounced differential response of members of the TLR family to ET3 delivered as protein versus by PLC could thus also be a key contributing factor to the generation of inhibitors in the former and their avoidance in the latter.

The PBMC from animals that received PLC-mcoET3 also exhibited differential expression (when compared to PBMC from animals receiving hFVIII/ET3 protein) of key transcripts involved in early B cell development, germinal center formation, development of germinal center B cells, and B cell affinity maturation, including downregulation of Interferon Regulatory Factor (IRF) 4 and of Nuclear Factor of Activated T Cells (NFATC1), and upregulation of B and T Lymphocyte Attenuator (BTLA) (69–71). As follicular T helper (Tfh) cells are known to be involved in the development of FVIII inhibitors (72, 73), and regulatory T cells (Tregs) in the induction of tolerance to FVIII (74), the present studies would have been further strengthened by performing flow cytometry on peripheral blood of the animals that received each FVIII “product”. Unfortunately, blood samples from these animals were not preserved in a manner that enabled us to perform these flow cytometric analyses, nor to isolate and sufficiently expand any FVIII-specific Tregs (74) that formed in these animals to allow further characterization.

In addition, we also found that exosomes produced by PLC-mcoET3 contain transcripts for proteins that are known to promote the generation of Tregs and/or the conversion of conventional T cells into Tregs such as TGF-β, CTLA-4, FLT3L, IL-10, IL-27, PDCD1/CD279, and HMOX-1, as well as transcripts for molecules that play a key role in the development of tolerogenic dendritic cells, including IDO1, FLT3L, IL-0, and IL-27. For instance, IDO1 and HMOX1 have specifically been associated with a lower incidence of FVIII inhibitors in people with hemophilia A (75). Exosomes also contained transcripts for HLA-E, HLA-F, and HLA-G, which are well-documented to promote immune-dampening/tolerance via multiple mechanisms, AIRE, which although normally associated with central tolerance, has also been shown to mediate deletional tolerance via extrathymic Aire-expressing cells (76), as well as PDCD1/CD279, which when activated by its ligand CD274/PDL-1 (also present within the exosomes) triggers apoptosis, thereby removing inhibitor-forming B cells. These analyses thus demonstrate the presence of transcripts for multiple proteins that are critical to a variety of pathways by which immune tolerance to FVIII could be induced by the transplanted mcoET3-PLC.

Surprisingly, administration of PLC-mcoET3 in animals with prior FVIII inhibitors resulted in an increase in the titer of these anti-FVIII/ET3 inhibitors; nonetheless, it is important to note that there was an increase in FVIII activity in 2 out of the 3 PLC-mcoET3 recipients. These data agree with a previous report on HA sheep harboring FVIII inhibitory antibodies, in which the administration of haploidentical (paternal) MSC transduced with a porcine FVIII-encoding lentivector did not reduce antibody titers but nonetheless resulted in the cessation of existent hemarthroses and all other spontaneous bleeding (77). While we would very much like to test this therapy in HA animals, as we previously described (78), these animals are time-consuming to generate, and they are extremely difficult to maintain, as they often die from spontaneous bleeds within only hours to days of birth if not treated with replacement factor. While these animals can be kept alive with human FVIII infusions, this quickly leads to the formation of relatively high-titer inhibitors (78), which would preclude, or at least greatly complicate, testing of this cell-based therapy. As a result, we have not yet had the opportunity to test this new therapy in the HA animals, but plan to do so in future studies.

In conclusion, the present studies demonstrate that the use of PLC as cellular vehicles allows the IV delivery of a highly immunogenic foreign FVIII molecule (ET3) to pediatric recipients, mediating long-term therapeutic plasma FVIII activity levels without triggering induction of inhibitors, thus highlighting the potential of this cell-based approach to treating HA. In addition, in-depth immune-focused gene expression analyses provide key insights into the very different response of the host immune system to FVIII/ET3 when delivered as a bolus infusion of protein versus when PLC expressing the protein are transplanted. The immune pathways differentially regulated between these delivery methods highlighted here could be leveraged to identify druggable targets to reduce, or perhaps eliminate, the risk of FVIII inhibitor induction following protein infusion.

## Data Availability

While no data were generated that require deposition into a public repository. All data will be made available upon request to the corresponding author, in compliance with Frontiers policy.
