# Supplementary material for: Transplanted gene-modified placental cells boost FVIII activity in pediatric sheep without eliciting immunity, toxicity, or adverse events
Source: Front Immunol. 2026 Jan 7;16:1716950. doi: 10.3389/fimmu.2025.1716950 (PMC12819314; doi:10.3389/fimmu.2025.1716950)
Supplement: Supplementary file 1 [file DataSheet1.pdf]

Supplementary Material

Supplementary Figures

Supplementary Figure 1

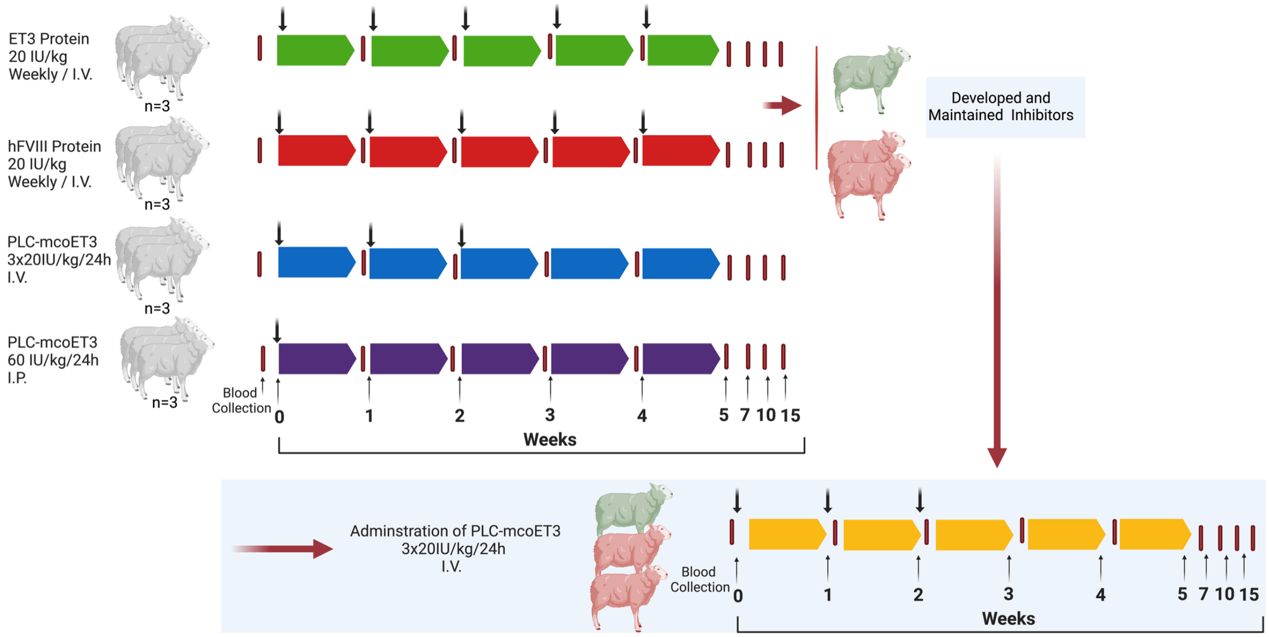

Supplementary Figure 1. Overview of study design. 12 juvenile sheep were administered one of the following FVIII-based treatments (n=3 animals per treatment): 1) purified ET3 protein – 20IU/kg given intravenously (IV) once per week for 5 weeks (shown in green); 2) purified human FVIII (hFVIII) protein – 20IU/kg given IV once per week for 5 weeks (shown in red); 3) human placental cells (PLC) transduced with a lentiviral vector encoding a myeloid codon-optimized ET3 transgene (PLC-mcoET3), given IV once per week for 3 weeks, at a cell dose calculated to provide 20IU/kg/24h (shown in blue); or 4) PLC-mcoET3 given as a single bolus injection intraperitoneally (IP) at a cell dose calculated to provide 60IU/kg/24h, to equal the total dose of PLC-mcoET3 given to the animals in the group that received PLC-mcoET3 via the IV route (shown in purple). Down-facing black arrows indicate times at which the purified ET3/hFVIII protein or the PLC-mcoET3 were administered. Vertical red bars and up-facing small black arrows indicate blood collections. Animals indicated with ### (n=3) received either purified ET3 protein or hFVIII protein and developed high-titer inhibitors. These animals were then used for the studies at the bottom grayed region of the figure (shown in yellow) to test whether administering PLC-mcoET3 could break pre-existing inhibitors and induce tolerance to ET3/hFVIII.

Supplementary Figure 2

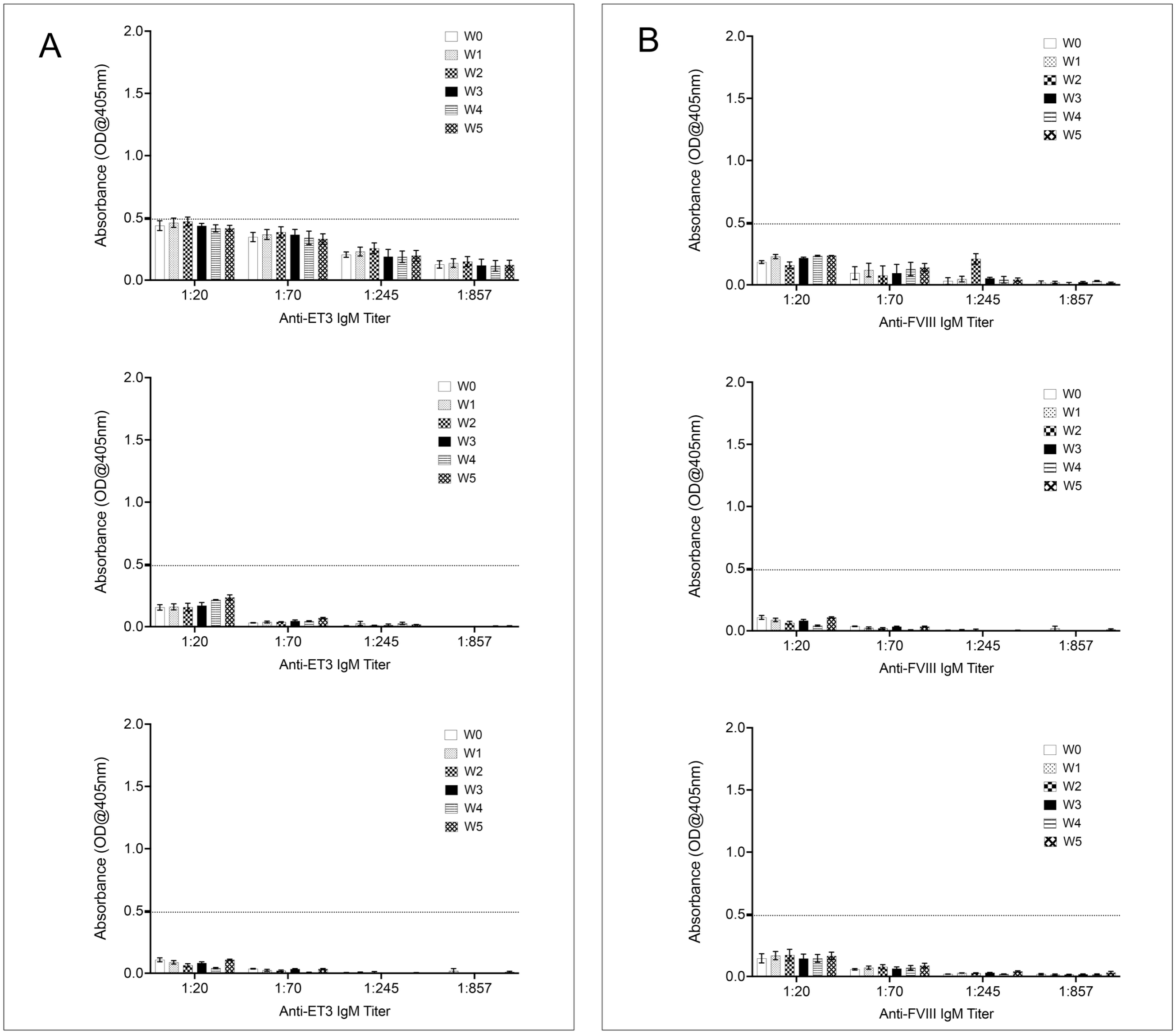

Supplementary Figure 2. Absence of IgM response in animals treated with purified ET3 or hFVIII protein. Serum was collected weekly from animals that received IV infusions of ET3 (n=3) or hFVIII (n=3) protein and assayed by ELISA for the presence of ET3-specific IgM antibodies. Neither the animals treated with ET3 (**A**) nor those treated with hFVIII (**B**) developed anti-ET3 IgMs. For each time point, ELISAs were performed in triplicate. Positive antibody titers were defined as the dilution of plasma with an absorbance value >2 SD above the mean OD from ELISAs performed with control sheep plasma (dotted horizontal line).

Supplementary Figure 3

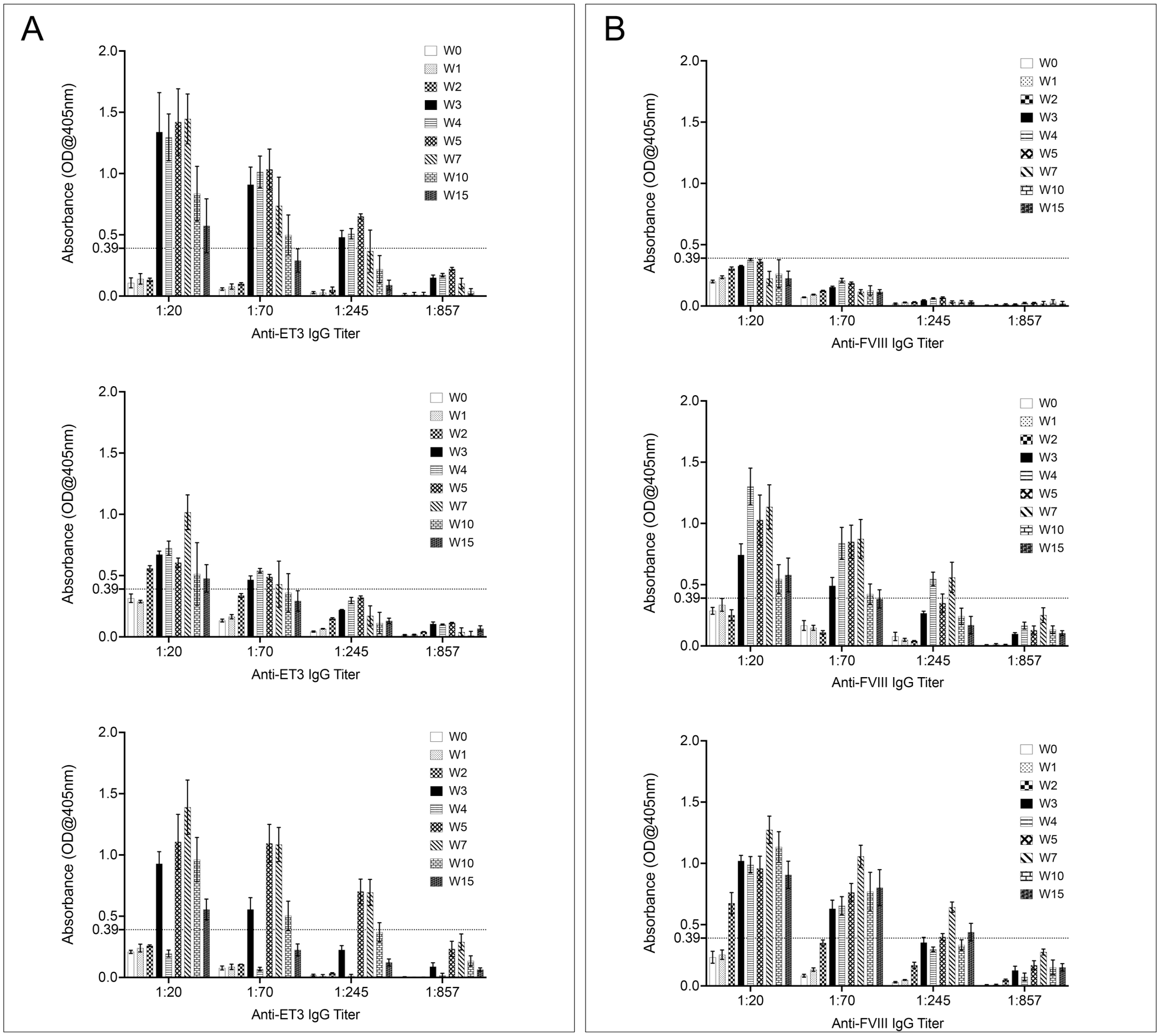

Supplementary Figure 3. Infusion of purified ET3 or hFVIII protein induces high-titer IgG response in juvenile animals. Serum was collected weekly from animals that received IV infusions of ET3 (n=3) or hFVIII (n=3) protein and assayed by ELISA for the presence of ET3-specific IgG antibodies. All animals treated with either ET3 (**A**) and 2 of the 3 animals treated with hFVIII (**B**) developed high-titer anti-ET3 IgGs. For each time point, ELISAs were performed in triplicate. Positive antibody titers were defined as the dilution of plasma with an absorbance value >2 SD above the mean OD from ELISAs performed with control sheep plasma (dotted horizontal line).

Supplementary Figure 4

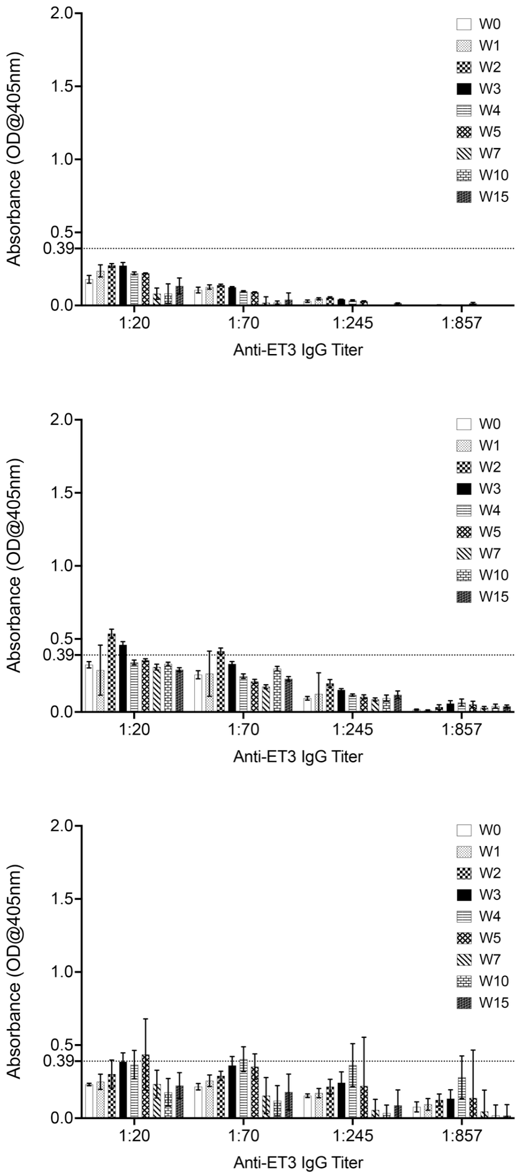

Supplementary Figure 4. Intraperitoneal (IP) infusion of PLC-mcoET3 to juvenile sheep results in very low titer anti-ET3 IgGs that disappear over time. Serum was collected weekly from animals that received a single IP infusion of PLC-mcoET3 (n=3) and assayed by ELISA for the presence of ET3-specific IgG antibodies. Two of the three animals treated with PLC-mcoET3 developed very low-titer anti-ET3 IgGs, while the third animal did not. However, these anti-ET3 IgGs disappeared from one animal by Week 4 after PLC-mcoET3 infusion and by Week 7 post-infusion in the other animal. For each time point, ELISAs were performed in triplicate. Positive antibody titers were defined as the dilution of plasma with an absorbance value >2 SD above the mean OD from ELISAs performed with control sheep plasma (dotted horizontal line).

Supplementary Figure 5.

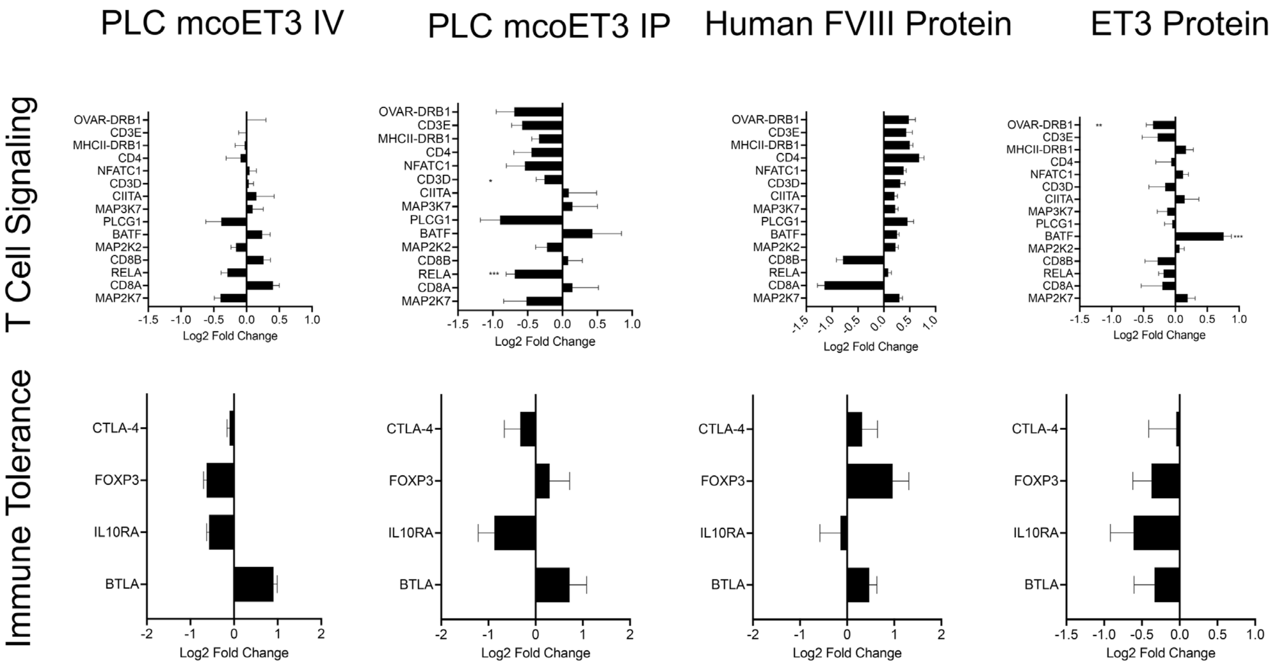

Supplementary Figure 5. T cell signaling and Immune tolerance gene expression. mRNA was isolated from PBMC collected from each of the animals at W0 as a base line, and at W5 after product administration. T cell signaling and immune tolerance related transcripts can be seen in recipients of PLC-mcoET3 IV and IP and in sheep who received FVIII and ET3 proteins.

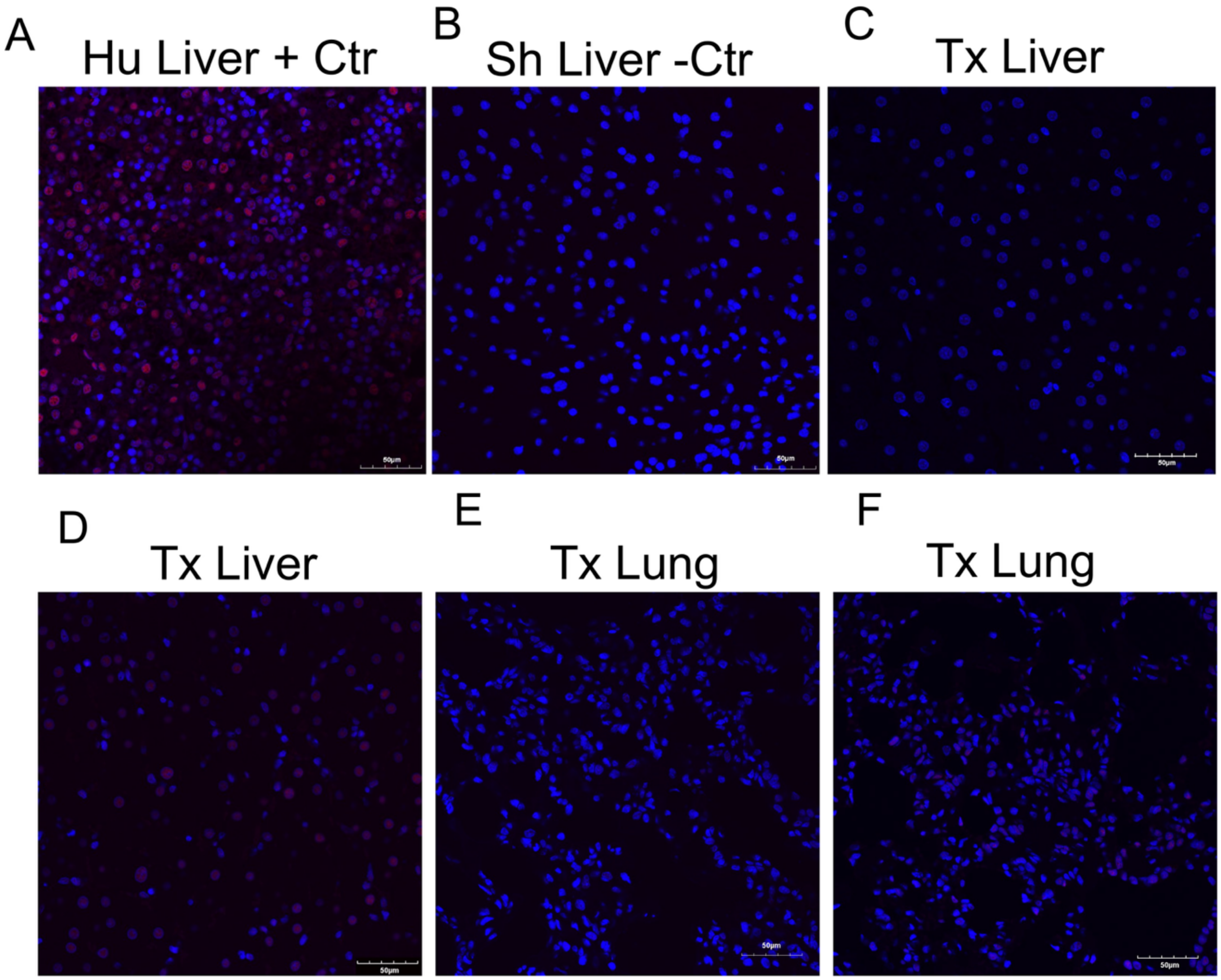

Supplementary Figure 6. Representative images of immunofluorescence with an antibody specific to the human nuclear antigen Ku80 (in red), showing direct visualization of engraftment of PLC-mcoET3 in the liver and lung of recipients. (A) DAPI in blue shows the nuclei of cells within the tissue and human nuclear antigen KU80 shows human cells in red; (B) Non-transplanted sheep liver demonstrating the specificity of the Ku80 staining to identify human cells; (C) No human cells were seen in this area of the right lobe of the liver in IP cell recipient 20002, but (D) a population of engrafted human cells were seen in the left lobe of the liver from this same animal; (E) No human cells were visualized in this area of the left inferior lobe of the lung in IV cell recipient 20007, but (F) a population of engrafted human cells were seen in the left superior lobe of the lung from this same animal. Confocal images were acquired with an Olympus Fluoview FV1000 confocal microscope and an Olympus UPlanFLN- 40x/1.30 oil objective
