# Supplementary material for: Transplanted gene-modified placental cells boost FVIII activity in pediatric sheep without eliciting immunity, toxicity, or adverse events
Source: Front Immunol. 2026 Jan 7;16:1716950. doi: 10.3389/fimmu.2025.1716950 (PMC12819314; doi:10.3389/fimmu.2025.1716950)
Supplement: Supplementary file 2 [file DataSheet2.pdf]

## Supplementary Tables

### Supplementary Table 1

NanoString multiplex gene expression assay gene target list for mRNA targets

| Gene Identifier | NCBI Accession # | Base Pair Position |
|---|---|---|
| ACTN1 | XM_004011053.1 | 1790-1889 |
| ADORA2A | XM_004023270.1 | 303-402 |
| AFDN | XM_027972665.1 | 2858-2957 |
| AHR | XM_004007775.4 | 1243-1342 |
| ATG7 | XM_004018530.1 | 742-841 |
| B2M | NM_001009284.2 | 276-375 |
| BATF | XM_004023592.4 | 628-727 |
| BATF3 | XM_004013897.3 | 2201-2300 |
| BCL6 | XM_004003049.4 | 9148-9247 |
| BLK | XM_004005333.1 | 544-643 |
| BTBD6 | XM_004023360.4 | 1168-1267 |
| BTLA | XM_004002916.1 | 629-728 |
| CCR7 | XM_004012868.4 | 1260-1359 |
| CD163 | ENSOART00000003108.1 | 1291-1390 |
| CD163L1 | NM_176651.1 | 1624-1723 |
| CD19 | XM_004020862.1 | 535-634 |
| CD244 | XM_004002675.3 | 1475-1574 |
| CD27 | XM_004006941.4 | 705-804 |
| CD274 | XM_004004362.4 | 360-459 |
| CD276 | XM_004010289.1 | 571-670 |
| CD28 | NM_001009441.1 | 190-289 |
| CD38 | XM_027971213.1 | 1282-1381 |
| CD3D | NM_001009382.1 | 502-601 |
| CD3E | NM_001009418.2 | 636-735 |
| CD3G | XM_004016069.3 | 521-620 |
| CD4 | NM_001129902.1 | 801-900 |
| CD40 | XM_004014616.4 | 290-389 |
| CD40LG | NM_001075101.1 | 274-373 |
| CD44 | XM_027979619.1 | 2136-2235 |
| CD6 | XM_004019839.1 | 1271-1370 |
| CD68 | XM_004012666.4 | 1071-1170 |
| CD69 | XM_004006885.4 | 454-553 |
| CD80 | NM_001024963.2 | 368-467 |
| CD84 | XM_004003717.3 | 477-576 |
| CD86 | NM_001038016.1 | 259-358 |
| CD8A | XM_004007263.1 | 552-651 |
| CD8B | XM_004007264.4 | 579-678 |
| CD9 | NM_001114764.1 | 646-745 |
| CD99 | XM_004022521.1 | 556-655 |
| CDC26 | XM_004003998.1 | 330-429 |
| CIITA | XM_015104076.2 | 3190-3289 |
| CLDN1 | NM_001185016.1 | 189-288 |

| | | |
|---|---|---|
| CLDN3 | **XM_004020967.4** | **846-945** |
| CLDN4 | NM_001185017.2 | 1048-1147 |
| CLDN7 | NM_001185018.1 | 344-443 |
| CPA3 | XM_004003271.4 | 329-428 |
| CSF3R | XM_004003483.3 | 2717-2816 |
| CTLA-4 | NM_001009214.1 | 223-322 |
| CTNNA1 | XM_004008841.4 | 1615-1714 |
| CTNND1 | XM_004016813.1 | 2045-2144 |
| CTSD | XM_027959254.1 | 1054-1153 |
| CTSW | XM_004019679.3 | 883-982 |
| CXCL12 | XM_004021573.3 | 232-331 |
| CXCR4 | NM_001277168.1 | 526-625 |
| CYBB | XM_004021996.4 | 2628-2727 |
| DLL1 | XM_012166492.2 | 884-983 |
| EOMES | XM_004018196.1 | 1568-1667 |
| ERAP2 | XM_004009561.1 | 1972-2071 |
| FCGR3A/B | NM_001139454.1 | 479-578 |
| FOXP3 | NM_001144947.1 | 574-673 |
| GATA3 | NM_001252183.1 | 930-1029 |
| GNLY | NM_001105262.1 | 359-458 |
| GZMA | XM_004016990.4 | 336-435 |
| HAVCR2 | XM_004009016.3 | 2159-2258 |
| HDC | XM_004010621.4 | 1273-1372 |
| HIF1A | XM_004010703.1 | 1502-1601 |
| HMBS | XM_012095568.3 | 699-798 |
| HPRT1 | XM_015105023.2 | 808-907 |
| HSD11B1 | NM_001009395.2 | 564-663 |
| ICAM1 | XM_027969187.1 | 1728-1827 |
| ICOS | XM_004004846.3 | 1783-1882 |
| IFNg | NM_001009803.1 | 461-560 |
| IKZF2 | XM_004004899.3 | 3516-3615 |
| IKZF4 | XM_004007470.1 | 1179-1278 |

| | | |
|---|---|---|
| IL10 | NM_001009327.1 | 473-572 |
| IL10RA | XM_004016061.1 | 1985-2084 |
| IL12A | NM_001009736.1 | 255-354 |
| IL12B | NM_001009438.1 | 605-704 |
| IL13 | NM_001082594.1 | 522-621 |
| IL17A | XM_004018887.4 | 100-199 |
| IL17F | XM_004018888.4 | 414-513 |
| IL1B | NM_001009465.2 | 630-729 |
| IL1R1 | XM_004006125.4 | 2169-2268 |
| IL2 | NM_001009806.1 | 273-372 |
| IL21 | NM_001256817.1 | 135-234 |
| IL21R | NM_001252180.1 | 536-635 |
| IL22 | XM_012159098.2 | 1760-1859 |
| IL23A | NM_001185122.2 | 247-346 |
| IL23R | XM_004003558.3 | 719-818 |
| IL2RA | NM_001009415.1 | 1386-1485 |
| IL21 | NM_001256817.1 | 135-234 |
| IL21R | NM_001252180.1 | 536-635 |
| IL22 | XM_012159098.2 | 1760-1859 |
| IL23A | NM_001185122.2 | 247-346 |
| IL23R | XM_004003558.3 | 719-818 |
| IL2RA | NM_001009415.1 | 1386-1485 |
| IL4 | NM_001009313.3 | 148-247 |
| IL4R | XM_004020855.1 | 1099-1198 |
| IL5 | NM_001009783.1 | 191-290 |
| IL6 | NM_001009392.1 | 412-511 |
| IRF4 | XM_004019154.1 | 634-733 |
| IRF7 | XM_027959960.1 | 577-676 |
| ITGAM | NM_001082593.1 | 1166-1265 |
| JAG1 | XM_004014644.1 | 3172-3271 |
| JAK1 | XM_004002047.1 | 3348-3447 |
| JAK2 | XM_004004357.3 | 3938-4037 |
| KLRB1 | XM_012170652.2 | 921-1020 |
| LAG3 | XM_004007581.1 | 729-828 |
| LNX1 | XM_004009828.4 | 1592-1691 |
| S100A12 | XM_004002522.3 | 262-361 |
| FCAR | XM_004015431.4 | 726-825 |

| | | |
|---|---|---|
| MAF | XM_015100144.1 | 1918-2017 |
| MAML2 | XM_004016533.1 | 2338-2437 |
| MAML3 | XM_004017239.1 | 834-933 |
| MAP2K2 | XM_004008603.4 | 764-863 |
| MAP2K7 | NM_001161879.1 | 754-853 |
| MAP3K7 | XM_004011267.4 | 1651-1750 |
| MAPK12 | XM_004023453.1 | 751-850 |
| MHCII-DRB1 | NM_001123402.1 | 704-803 |
| MMP9 | XM_004014614.4 | 604-703 |
| MS4A1 | XM_004016513.4 | 727-826 |
| MS4A2 | XM_004016510.4 | 1136-1235 |
| MTOR | NM_001145455.1 | 5682-5781 |
| NCR1 | NM_001199068.1 | 290-389 |
| NFATC1 | XM_004020586.1 | 1656-1755 |
| NKG7 | XM_004015407.3 | 689-788 |
| NOTCH1 | XM_004023154.1 | 1604-1703 |
| NOTCH2 | XM_004002392.4 | 7841-7940 |
| OVAR-DRB1 | NM_001280698.1 | 139-238 |
| PDCD1 | XM_004001756.1 | 523-622 |
| PDCD1LG2 | XM_004005308.1 | 519-618 |
| PECAM1 | XM_004013029.4 | 1755-1854 |
| PLCG1 | XM_004014836.1 | 1824-1923 |
| PNOC | XM_004004438.4 | 360-459 |
| PRF1 | XM_004021436.4 | 574-673 |
| PSMA2 | XM_004007997.4 | 689-788 |
| PSMA3 | XM_004010681.4 | 458-557 |
| PSMA6 | NM_001139446.2 | 456-555 |
| PTGDR2 | XM_004019836.1 | 556-655 |
| PTGER4 | XM_004017023.1 | 875-974 |
| PTPRC | XM_004013932.3 | 2026-2125 |
| RELA | XM_004019901.1 | 877-976 |
| RUNX3 | XM_004005511.1 | 311-410 |
| SEC22B | XM_004002393.4 | 1094-1193 |
| SELL (CD62L) | XM_004013677.4 | 871-970 |
| SH2D1A | XM_004022366.4 | 430-529 |
| SMAD2 | XM_004020532.1 | 1028-1127 |

| | | |
|---|---|---|
| SMAD3 | XM_004010875.1 | 1287-1386 |
| SMURF1 | XM_004021292.1 | 1341-1440 |
| SPIB | XM_012157885.2 | 358-457 |
| STAT1 | NM_001166203.1 | 803-902 |
| STAT3 | XM_004012925.1 | 2072-2171 |
| STAT4 | XM_004004773.2 | 951-1050 |
| STAT5A | NM_001009402.2 | 1481-1580 |
| STAT5B | XM_004012924.2 | 1133-1232 |
| STAT6 | XM_004006555.3 | 2511-2610 |
| TBX21 | NM_013351.2 | 1133-1232 |
| TCL1A | XM_004018143.1 | 4-103 |
| TGFB1 | NM_001009400.2 | 675-774 |
| TIGIT | XM_004003792.1 | 274-373 |
| TLR2 | NM_001048231.1 | 1094-1193 |
| TLR3 | NM_001135928.1 | 727-826 |
| TLR4 | NM_001135930.1 | 1847-1946 |
| TLR7 | NM_001135059.1 | 1340-1439 |
| TLR8 | NM_001135929.1 | 1879-1978 |
| TLR9 | NM_001011555.1 | 2236-2335 |
| TNF | NM_001024860.1 | 928-1027 |
| TNFRSF17 | XM_004020779.1 | 410-509 |
| TYK2 | XM_004009295.1 | 2500-2599 |
| UBA5 | XM_004003335.4 | 1743-1842 |
| VSIR | XM_004021444.4 | 1168-1267 |
| XCL1/2 | NM_001009427.1 | 203-302 |
| ZAP70 | XM_004007340.1 | 120-219 |
| ZBTB16 | XM_004016559.1 | 552-651 |

# **Supplementary Table 2**:

## Summary of upregulated endothelial- and hepatocyte-specific genes in Liver

| | | | Animal Number | | | | | | | | |
|---|---|---|---|---|---|---|---|---|---|---|---|
| | | | PLC IP | | | PLC IV | | | Protein + PLC IV | | |
| **Name** | **Ensembl ID** | **Protein** | **20001** | **20002** | **20002 #2** | **19005** | **20006** | **20007** | **19003** | **20003** | **20004** |
| PROX1 | ENSG00000117707 | prospero homeobox 1 | | + | + | + | | + | | + | + |
| CD36 | ENSG00000135218 | Platelet glycoprotein IV | | + | + | + | + | + | | + | |
| MRC1 | ENSG00000260314 | Mannose receptor C-type 1 | + | + | | + | | + | | + | |
| LYVE1 | ENSG00000133800 | Lymphatic vessel endothelial hyaluronan receptor 1 | + | | | | | | | + | |
| VWF | ENSG00000110799 | von Willebrand Factor | + | | + | | | | | | |
| F8 | ENSG00000185010 | Coagulation Factor VIII | + | + | + | + | | + | | + | |
| MRC2 | ENSG00000011028 | Mannose receptor C type 2 | | | | | | | | | |
| PECAM1 | ENSG00000261371 | PECAM 1 / CD31 | + | + | | | | | | | |
| ICAM1 | ENSG00000090339 | Intercellular adhesion molecule 1 | | | | | | | | | |
| | | | | | | | | | | | |
| F9 | ENSG00000101981 | Coagulation Factor IX | + | + | + | + | + | + | | + | |
| MASP2 | ENSG00000009724 | MBL associated serine protease 2 | | + | + | + | | + | | + | |
| LEPR | ENSG00000116678 | Leptin receptor | + | + | + | + | | + | | + | + |
| CRP | ENSG00000132693 | C-reactive protein | | | | | | | | + | |
| APOA2 | ENSG00000158874 | Apolipoprotein A2 | | | | | | | | | |
| F5 | ENSG00000198734 | Coagulation Factor V | | + | + | + | + | + | | + | + |
| PROX1 | ENSG00000117707 | prospero homeobox 1 | | + | + | + | | + | | + | + |
| APOB | ENSG00000084674 | Apolipoprotein B | | + | + | + | | | | + | |
| CP | ENSG00000047457 | Ceruloplasmin | | + | + | + | | | | + | |
| ALB | ENSG00000163631 | Albumin | | + | + | + | | + | | + | |
| AFP | ENSG00000081051 | Alpha-fetoprotein | | | | | | | | | |
| FGB | ENSG00000171564 | Fibrinogen beta chain | | + | + | + | | + | | + | |
| FGA | ENSG00000171560 | Fibrinogen alpha chain | | + | + | + | + | + | | + | |
| FGG | ENSG00000171557 | Fibrinogen gamma chain | | + | + | + | | + | | + | |
| APOM | ENSG00000204444 | Apolipoprotein M | | + | + | + | + | + | | + | |
| GSTA2 | ENSG00000244067 | Glutathione S-transferase alpha 2 | | + | + | + | + | + | | + | |
| CYP3A7 | ENSG00000160870 | Cytochrome P450 family 3 subfamily A member 7 | | | | | | | | | |
| CYP3A4 | ENSG00000160868 | Cytochrome P450 family 3 subfamily A member 4 | | | | | | | | + | |
| TFR2 | ENSG00000106327 | Transferrin receptor 2 | + | | + | + | | + | | + | |
| FGL1 | ENSG00000104760 | Fibrinogen like 1 | | + | + | + | + | + | | + | |
| CYP7A1 | ENSG00000167910 | Cytochrome P450 family 7 subfamily A member 1 | | | | + | + | + | | | |
| HHEX | ENSG00000152804 | Hematopoietically expressed homeobox | | | | + | | | | | |
| CYP2C9 | ENSG00000138109 | Cytochrome P450 family 2 subfamily C member 9 | | | | + | | | | + | |
| CYP2C8 | ENSG00000138115 | Cytochrome P450 family 2 subfamily C member 8 | | + | | + | | | | | |
| CYP2E1 | ENSG00000130649 | Cytochrome P450 family 2 subfamily E member 1 | | + | + | + | + | + | | + | |
| LRP5 | ENSG00000162337 | LDL receptor related protein 5 | | | | + | | | | | |
| SERPINH1 | ENSG00000149257 | Serpin family H member 1 | | | | | | | | | |
| APOA1 | ENSG00000118137 | Apolipoprotein A1 | | | | + | | | | + | |
| SERPINA10 | ENSG00000140093 | Serpin family A member 10 | | + | + | + | | | | + | |
| SERPINA6 | ENSG00000170099 | Serpin family A member 6 | | | | | | | | | |
| AQP9 | ENSG00000103569 | Aquaporin 9 | | + | | + | | | | + | |
| CYP1A1 | ENSG00000140465 | Cytochrome P450 family 1 subfamily A member 1 | | + | + | + | + | + | | + | |
| CYP1A2 | ENSG00000140505 | Cytochrome P450 family 1 subfamily A member 2 | | | + | + | | + | | + | |
| TAT | ENSG00000198650 | Tyrosine aminotransferase | | + | + | + | + | + | | + | |
| ASGR1 | ENSG00000141505 | Asialoglycoprotein receptor 1 | | | + | | + | | | + | |
| APOH | ENSG00000091583 | Apolipoprotein H | | + | + | + | + | + | | + | + |
| TTR | ENSG00000118271 | Transthyretin | | + | + | + | + | + | | + | + |
| CEBPA | ENSG00000245848 | CCAAT enhancer binding protein alpha | | | | + | | | | | |
| CYP2A6 | ENSG00000255974 | Cytochrome P450 family 2 subfamily A member 6 | | | + | + | | + | | + | |
| CYP2A7 | ENSG00000198077 | Cytochrome P450 family 2 subfamily A member 7 | | | + | | + | | | | |
| CYP2B6 | ENSG00000197408 | Cytochrome P450 family 2 subfamily B member 6 | + | | + | + | | | | + | |
| HNF4A | ENSG00000101076 | Hepatocyte nuclear factor 4 alpha | + | + | + | + | + | + | | + | |
| PCK1 | ENSG00000124253 | Phosphoenolpyruvate carboxykinase 1 | + | + | + | + | + | + | | + | |
| CYP2D6 | ENSG00000100197 | Cytochrome P450 family 2 subfamily D member 6 | | | | + | | | | | |
| OTC | ENSG00000036473 | Ornithine transcarbamylase | | + | + | + | | + | | + | + |

## **Supplementary Table 3**: Summary of upregulated genes in Lung

| | | | Animal Number | | | | | | | | |
|---|---|---|---|---|---|---|---|---|---|---|---|
| | | | PLC IP | | | PLC IV | | | Protein + PLC IV | | |
| Gene Name | Ensembl ID | Protein | 20001 | 20002 | 20002 #2 | 19005 | 20006 | 20007 | 19003 | 20003 | 20004 |
| AQP1 | ENSG00000240583 | Aquaporin 1 | + | + | + | + | + | + | + | + | |
| AQP3 | ENSG00000165272 | Aquaporin 3 | | | | | | | | | |
| AQP4 | ENSG00000171885 | Aquaporin 4 | | | | | | | | | |
| AQP5 | ENSG00000161798 | Aquaporin 5 | | | + | | + | | | | |
| CLDN1 | ENSG00000163347 | Claudin 1 | | + | | + | + | | | + | |
| CLDN3 | ENSG00000165215 | Claudin 3 | | + | | | + | | | | |
| CLDN4 | ENSG00000189143 | Claudin 4 | | | | | + | | | | |
| CLDN7 | ENSG00000181885 | Claudin 7 | | | | | | | | | |
| CLDN18 | ENSG00000066405 | Claudin 18 | + | + | + | + | + | + | + | + | + |
| SCNN1B | ENSG00000168447 | Sodium channel epithelial 1 subunit beta | + | + | + | | + | + | + | + | |
| ATP1A1 | ENSG00000163399 | ATPase Na+/K+ transporting subunit alpha 1 | + | + | + | + | + | + | + | + | |
| SFTPC | ENSG00000168484 | Surfactant protein C | + | | + | + | + | + | + | + | |
| ABCA3 | ENSG00000167972 | ATP binding cassette subfamily A member 3 | + | + | + | + | + | + | + | + | |
| CAV1 | ENSG00000105974 | Caveolin 1 | | | | | | | | | |
| CDH1 | ENSG00000039068 | Cadherin 1 | + | + | + | + | + | + | + | + | |
| CDH2 | ENSG00000170558 | Cadherin 2 | | | | | | | | | |
| gp36/LMAN2 | ENSG00000169223 | lectin, mannose binding 2 | + | | + | | + | | + | | |
| AGER | ENSG00000204305 | receptor specific for advanced glycosylation end-products | + | + | + | + | + | + | + | + | + |
| CFTR | ENSG00000001626 | CF transmembrane conductance regulator | + | | + | + | + | | | + | |

## **Supplementary Table 4**: Summary of upregulated genes in Thymus

| | | | Animal Number | | | | | | |
|---|---|---|---|---|---|---|---|---|---|
| | | | PLC IP | | PLC IV | | Protein + PLC IV | | |
| Thymic Marker | Ensembl ID | Protein Product | 19005 | 19004 | 20006 | 20007 | 19003 | 20003 | 20004 |
| KRT5 | ENSG00000186081 | Cytokeratin 5 | + | + | + | | | + | |
| KRT8 | ENSG00000170421 | Cytokeratin 8 | | | | | | | |
| KRT13 | ENSG00000171401 | Cytokeratin 13 | | | | | | | |
| F8 | ENSG00000185010 | Coagulation Factor VIII | | | + | | + | | |
| AIRE | ENSG00000160224 | AIRE | | | | | | | |
| PDPN | ENSG00000162493 | Podoplanin | | | | | | | |
| CD200/MOX1/MOX2 | ENSG00000091972 | Immunoregulatory interactions between a Lymphoid & non-Lymphoid cell | | + | + | | | + | |
| COL4A3 | ENSG00000169031 | | | | | | | | |
| EpCAM | ENSG00000119888 | epithelial cell adhesion molecule | | | | | | | |
| FOXN1 | ENSG00000109101 | Forkhead box N1 | | + | + | | | + | |
| ASCL1 | ENSG00000139352 | Achaete-scute family bHLH transcription factor 1 | | | | | | | |
| INSM1 | ENSG00000173404 | INSM transcriptional repressor 1 | + | | | | | | |
| EHF | ENSG00000135373 | ETS homologous factor | | | | | | | |
| POU2F3 | ENSG00000137709 | POU class 2 homeobox 3 | | | | | | | |
| SPIB | ENSG00000269404 | Spi-B transcription factor | | | | | | + | |
| CLDN3 | ENSG00000165215 | Claudin 3 | | | | | | | |
| CLDN4 | ENSG00000189143 | Claudin 4 | | | | | | | |
